# Activation of Nrf2 in keratinocytes causes chloracne (MADISH)-like skin disease in mice

**DOI:** 10.1002/emmm.201303281

**Published:** 2014-02-06

**Authors:** Matthias Schäfer, Ann-Helen Willrodt, Svitlana Kurinna, Andrea S Link, Hany Farwanah, Alexandra Geusau, Florian Gruber, Olivier Sorg, Aaron J Huebner, Dennis R Roop, Konrad Sandhoff, Jean-Hilaire Saurat, Erwin Tschachler, Marlon R Schneider, Lutz Langbein, Wilhelm Bloch, Hans-Dietmar Beer, Sabine Werner

**Affiliations:** 1 Department of Biology, Institute of Molecular Health SciencesETH Zurich, Zurich, Switzerland; 2 Institute for Physiology and Pathophysiology, University of ErlangenErlangen, Germany; 3 LIMES, Membrane Biology & Lipid Biochemistry Unit, c/o Kekulé-Institute for Organic Chemistry and Biochemistry, University of BonnBonn, Germany; 4 Department of Dermatology, Medical University of ViennaVienna, Austria; 5 Swiss Centre for Applied Human Toxicology, Dermatotoxicology Unit, University of GenevaGeneva, Switzerland; 6 Department of Dermatology, School of Medicine, University of Colorado DenverDenver, CO, USA; 7 Institute of Molecular Animal Breeding and BiotechnologyLMU Munich, Munich, Germany; 8 Department of Genetics of Skin Carcinogenesis, German Cancer Research CenterHeidelberg, Germany; 9 Department of Molecular and Cellular Sport Medicine, German Sport University CologneCologne, Germany; 10 Department of Dermatology, University Hospital ZurichZurich, Switzerland

**Keywords:** acne, sebaceous gland, Nrf2, oxidative stress, skin

## Abstract

The transcription factor Nrf2 is a key regulator of the cellular stress response, and pharmacological Nrf2 activation is a promising strategy for skin protection and cancer prevention. We show here that prolonged Nrf2 activation in keratinocytes causes sebaceous gland enlargement and seborrhea in mice due to upregulation of the growth factor epigen, which we identified as a novel Nrf2 target. This was accompanied by thickening and hyperkeratosis of hair follicle infundibula. These abnormalities caused dilatation of infundibula, hair loss, and cyst development upon aging. Upregulation of epigen, secretory leukocyte peptidase inhibitor (Slpi), and small proline-rich protein 2d (Sprr2d) in hair follicles was identified as the likely cause of infundibular acanthosis, hyperkeratosis, and cyst formation. These alterations were highly reminiscent to the phenotype of chloracne/“metabolizing acquired dioxin-induced skin hamartomas” (MADISH) patients. Indeed, SLPI, SPRR2, and epigen were strongly expressed in cysts of MADISH patients and upregulated by dioxin in human keratinocytes in an NRF2-dependent manner. These results identify novel Nrf2 activities in the pilosebaceous unit and point to a role of NRF2 in MADISH pathogenesis.

## Introduction

The skin functions as a barrier, which protects our body from harmful environmental insults, including microorganisms, UV light, and toxic chemicals. Many of them lead to the formation of reactive oxygen species (ROS), which at high concentrations damage cellular macromolecules. ROS-induced damage is therefore involved in skin tumor formation, aging, and in the pathogenesis of inflammatory skin diseases, such as psoriasis, atopic dermatitis, and contact dermatitis.

The skin has evolved effective mechanisms for the protection from ROS damage, including ROS detoxification and DNA repair. A master regulator of the cellular antioxidant defense is nuclear factor erythroid derived 2, like 2 (Nrf2). This transcription factor induces expression of various genes involved in the cellular redox balance, including the genes encoding glutamate cysteine ligase modifier (Gclm) and catalytic (Gclc) subunits, phase II detoxifying enzymes, such as NAD(P)H dehydrogenase, quinone 1 (Nqo1), and transporters, including multidrug resistance proteins (Mrps).

Nrf2 expression and activity form a basal to suprabasal gradient in the murine epidermis, which contributes to an intra-epidermal cytoprotection gradient. This results in strong protection of the uppermost epidermal layers from ROS-induced damage (Schäfer *et al*, 2010; Piao *et al*, 2012). Nrf2 gets further activated by electrophilic compounds, which directly bind and inactivate the Nrf2 inhibitor protein Keap1 (Tong *et al*, 2006).

Due to its central role in ROS detoxification, Nrf2 is an attractive target for pharmacological protection of the skin. A variety of Nrf2 activators, including sulforaphane, tert-butyl-hydroquinone (tBHQ), and resveratrol, have been discovered, which are antioxidant supplements of cosmetic products. Some of these compounds protected cultured keratinocytes from damage induced by UV irradiation or treatment with toxic chemicals (Gao *et al*, 2001; Liu *et al*, 2011; Lieder *et al*, 2012). *In vivo,* short-term topical sulforaphane treatment protected skin from acute UV toxicity and from chemically and UV-induced carcinogenesis (Dinkova-Kostova *et al*, 2006; Gills *et al*, 2006). Furthermore, UVB-induced ROS damage and subsequent keratinocyte apoptosis were reduced in transgenic mice expressing a constitutively active Nrf2 (caNrf2) mutant in keratinocytes (Schäfer *et al*, 2010).

However, there is increasing evidence for harmful consequences of Nrf2 activation in the skin. A variety of cysteine-reactive skin sensitizers, such as oxazolone or 2,4-dinitrofluorobenzene (DNFB), activate Nrf2, which contributes to their skin sensitization potential (Natsch, 2010). Furthermore, long-term Nrf2 activation in murine skin through deletion of the Nrf2 inhibitor Keap1 caused hyperkeratosis (Wakabayashi *et al*, 2003). Even more worrisome is the correlation of stabilized Nrf2 with increased malignancy and chemoresistance of tumor cells (Sporn & Liby, 2012). This is important for the skin, since Nrf2 activating mutations have been identified in human cutaneous squamous cell carcinomas (Kim *et al*, 2010). It is therefore essential to analyze the harmful consequences of Nrf2 activation and to understand the underlying mechanisms.

Genetic and pharmacological Nrf2 activation in murine keratinocytes *in vivo* induced severe acanthosis and hyperkeratosis strongly resembling ichthyosis in humans (Schäfer *et al*, 2012). This was caused by Nrf2-induced overexpression of secretory leukocyte peptidase inhibitor (Slpi) and of the small proline-rich protein 2d (Sprr2d) in the epidermis. These two proteins normally contribute to the ROS-protective and anti-inflammatory activities of Nrf2 (Ishii *et al*, 2005; Vermeij *et al*, 2011). However, elevated expression of Slpi impaired the desquamation of the *stratum corneum,* most likely due to inhibition of the proteinase kallikrein 7, which degrades corneodesmosomes. The enhanced levels of the cornified envelope protein Sprr2d caused corneocyte fragility, resulting in a barrier defect and subsequent mild inflammation and keratinocyte hyperproliferation (Schäfer *et al*, 2012).

Here we used a genetic and a pharmacological strategy to identify novel effects of Nrf2 on the pilosebaceous unit, and we identified responsible target genes. Importantly, the abnormalities observed upon Nrf2 activation in mice strongly resemble the pathology seen in patients with chloracne/metabolizing acquired dioxin-induced skin hamartomas (MADISH; Saurat & Sorg, 2010) at the histological and molecular level and point to a role of activated NRF2 in the pathogenesis of this disorder.

## Results

### Nrf2 activation in keratinocytes causes sebaceous gland hypertrophy and sebum congestion

**Figure 1:**
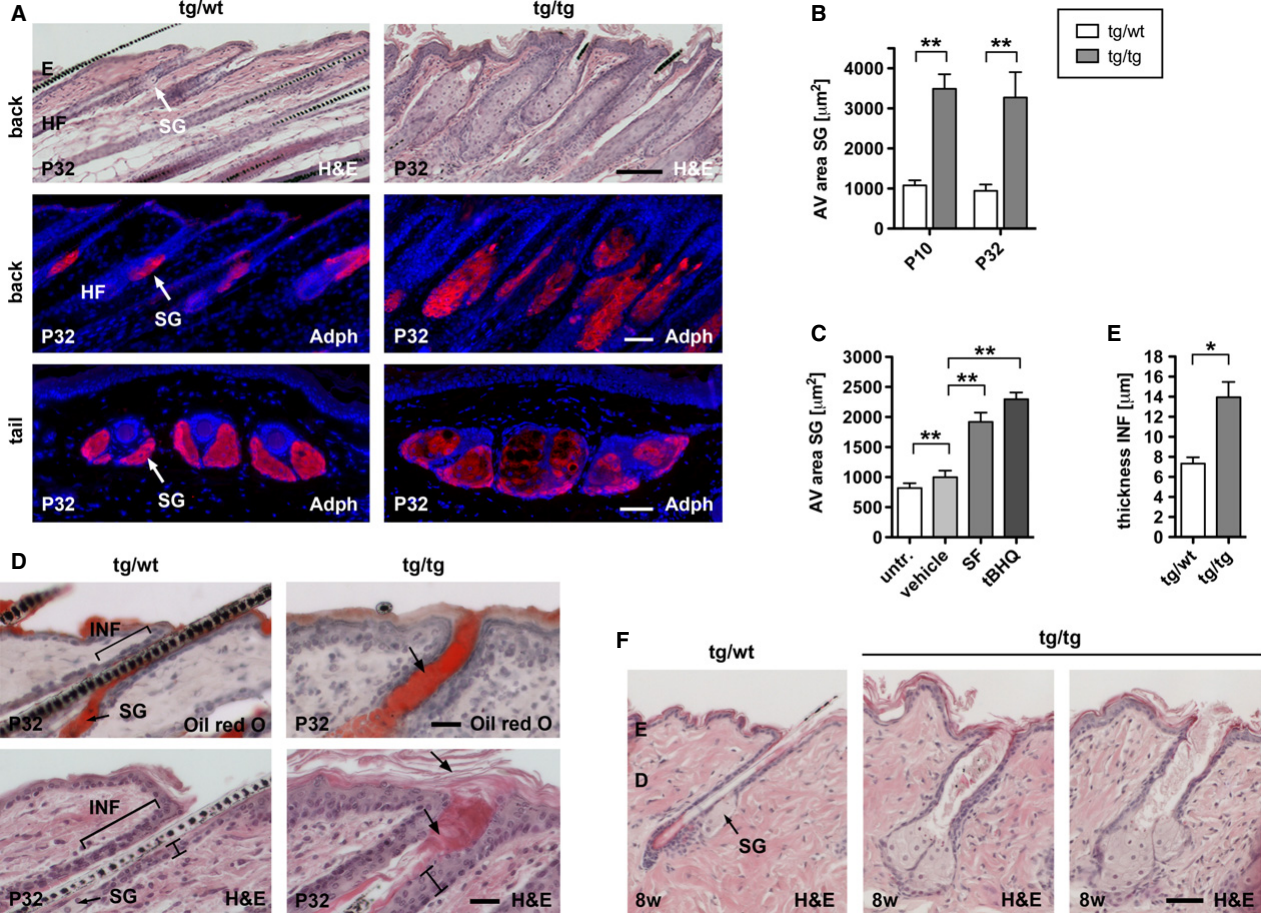
Morphology of sebaceous glands and infundibula in K5cre-CMVcaNrf2 mice.
Upper panel: H&E staining of longitudinal back skin sections. Scale bar: 100 μm. Middle and lower panel: immunofluorescence staining for Adph on longitudinal back skin (middle panel) and transverse tail skin sections (lower panel) of P32 tg/wt and tg/tg mice. Note larger SG in tg/tg back and tail. Scale bar: 50 μm.Back skin SG area in P10 (*N* = 7/6, **P = 0.0012) and P32 (*N* = 5/7, **P = 0.0025) tg/wt and tg/tg mice.Morphometrical analysis of SG area of untreated, vehicle (*N* = 10/6, **P = 0.0017), sulforaphane (*N* = 6, **P = 0.0022), and tBHQ (*N* = 6, **P = 0.0022) treated wt mice.Oil Red O (upper panel) and H&E staining (lower panel) of longitudinal back skin sections of P32 tg/wt and tg/tg mice. In tg/tg mice, infundibula (INF) are filled with sebum (indicated by arrow in upper panel), thickened (indicated by bar in lower panel), and hyperkeratotic (indicated by arrows in lower panel). Scale bars: 25 μm.Average thickness of infundibula (*N* = 5, *P = 0.0119) of tg/wt and tg/tg mice.H&E staining of longitudinal back skin sections of 8 w tg/wt and tg/tg mice. Note dilatation of infundibula in tg/tg mice. Scale bar: 50 μm. Data information: Values are shown as the mean with s.d. All *P*-values were calculated by Mann–Whitney *U*-test. AV, average; E, epidermis; D, dermis; HF, hair follicle; INF, infundibulum; SG, sebaceous gland.

We previously generated transgenic mice expressing a caNrf2 mutant under control of a β-actin promoter and CMV enhancer in keratinocytes using a strategy that allows expression of the transgene in the presence of Cre recombinase. For expression of caNrf2 in all keratinocytes, mice expressing Cre under the control of the keratin 5 (K5) promoter were used (Ramirez *et al*, 2004). The double transgenic mice, designated K5cre-CMVcaNrf2 mice, are characterized by acanthosis and severe hyperkeratosis in the epidermis (Schäfer *et al*, 2012). Interestingly, histological analyses and immunofluorescence staining for perilipin 2 (adipophilin, Adph) showed that the sebaceous glands (SG) in back and tail skin (Fig 1A,B) and the meibomian glands of the eyelids (Supplementary Fig 1A) are strongly enlarged in K5cre-CMVcaNrf2 mice. Consistent with these findings, their tail skin was oily and sticky, and the eyes were wet and sometimes partially closed, suggesting sebum/meibum overproduction (seborrhea).

Repeated topical treatment with the Nrf2-activating compounds sulforaphane or tert-butyl hydroquinone (tBHQ) (Schäfer *et al*, 2012) also caused enlargement of SGs in wild-type mice (Fig 1C), but not in *Nrf2* ko mice (Supplementary Fig 1B), demonstrating that activation of endogenous Nrf2 has a similar effect as the *caNrf2* transgene.

**Figure 2:**
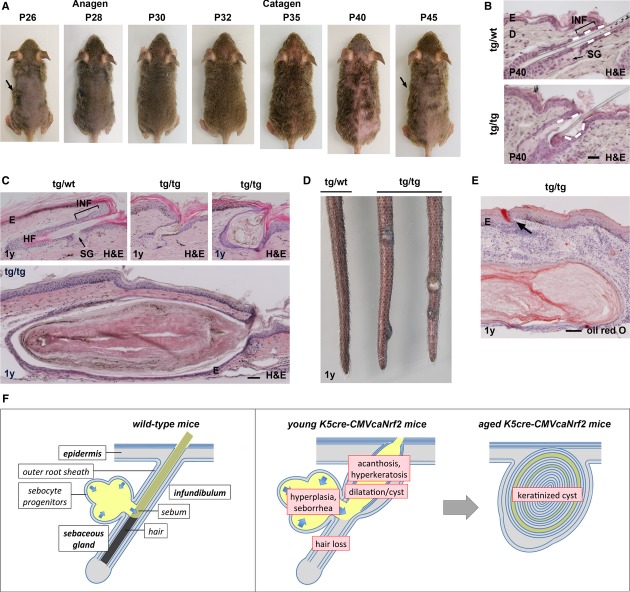
Cyclic hair loss and cutaneous cyst formation in K5cre-CMVcaNrf2 mice.
Tg/tg mouse at P26, P28, P30, P32, P35, P40, and P45. Note visible hair growth at P26 to P32 (anagen) and hair loss at P35 and P40 (catagen). Arrows point to the same islet of hairs at P26 and P45.H&E staining of longitudinal back skin sections of P40 tg/wt and tg/tg mice. Note widened infundibulum (INF) and reduced anchorage of hair (indicated by dashed line in upper panel). Scale bar: 50 μm.H&E staining of longitudinal tail skin sections of 1-year (1 y)-old mice showing dilatation (indicated by arrow in left panel) and cysts (right panel) in the tail skin of tg/tg mice. Scale bar: 50 μm.Tail skin of 1-y-old tg/wt and tg/tg mice. Note large cysts in tg/tg mice.Oil red O staining of a large tail skin cyst. Note ring-shaped weak oil red O staining. Arrow points to sebum in a hair follicle. Scale bar: 100 μm.Model of the pilosebaceous phenotype in K5cre-CMVcaNrf2 mice. Left panel: scheme of the pilosebaceous unit of wild-type skin. Arrows indicate sebocyte differentiation and sebum flow (yellow) in the SG. Middle panel: scheme of the pilosebaceous unit of young K5cre-CMVcaNrf2 mice. SG hyperplasia causes seborrhea. Acanthosis and hyperkeratosis of infundibula as well as hyperkeratosis of the interfollicular epidermis impair sebum outflow. Together, this results in dilatation of infundibula. Consequently, the hair anchorage is reduced, resulting in hair loss. Right panel: In aged K5cre-CMVcaNrf2 mice, continuous keratinization leads to formation of keratinized cutaneous cysts.

In addition to the SG abnormalities, K5cre-CMVcaNrf2 mice had dilated hair follicle infundibula (Fig 1D, upper and lower panel). Oil Red O staining revealed the presence of sebum in the widened infund-ibula of K5cre-CMVcaNrf2 mice (Fig 1D, upper panel), which were characterized by strong acanthosis and hyperkeratosis (Fig 1D, lower panel and 1E). Together, hyperkeratosis of infundibula and the interfollicular epidermis subsequently form a mechanical barrier, which prevent sebum outflow. The dilatation of the infundibulum was even more severe in hair follicles without a hair, most likely because the hair prevented a complete occlusion of the hair follicle by the hyperkeratotic *stratum corneum* and thus enabled sebum outflow (Fig 1F). Hyperkeratosis, in combination with sebum congestion, lead to keratin and sebum accumulation, and the resulting pressure finally caused dilatation of hair follicle infundibula (see scheme in Fig 2F).

### K5cre-CMVcaNrf2 mice exhibit cyclic hair loss and malformed hairs

In addition to the SG abnormalities, K5cre-CMVcaNrf2 mice exhibited cyclic hair loss, resulting in patchy baldness during catagen and telogen (Fig 2A and Supplementary Fig 1C–E). This was not due to a reduction in the number of hair follicles in the affected areas (Supplementary Fig 1F). Moreover, the follicles underwent all phases of the hair cycle, although the hair cycle of transgenic mice preceded the cycle of control mice by approximately 4 days (Supplementary Fig 2). Anagen hair follicles had a normal length, and follicles included a hair. Immunostaining of keratins expressed in various root sheath compartments revealed no obvious defects in differentiation of lower hair follicle keratinocytes (root sheath) and trichocytes (hair fiber) during hair formation (Supplementary Fig 3). Thus, hair follicles of K5cre-CMVcaNrf2 mice undergo normal differentiation and obviously form a functional hair during anagen, which, however, is lost prematurely during catagen or telogen. Interestingly, telogen pelage hairs were only anchored in the lower part of hair follicles due to dilation of infundibula (Fig 2B), suggesting that the hair loss during catagen and telogen is the consequence of reduced anchorage of pelage hairs.

The remaining pelage hair as well as the whiskers of K5cre-CMVcaNrf2 mice appeared shaggy and wavy (Supplementary Fig 1G). Furthermore, the majority of pelage hairs was thinner and curly (Supplementary Fig 1H) and had spiky cuticle cells (Supplementary Fig 1J), which explains the shaggy appearance of the remaining fur. These malformations of the remaining hairs are most likely the consequence of the narrowing of the hair canal due to infundibular hyperkeratosis and acanthosis.

### Nrf2 activation affects hair follicles and sebaceous glands in a dose-dependent manner

In K5cre-caNrf2 mice, caNrf2 is also under control of a β-actin promoter, but not of a CMV enhancer, resulting in a weaker activation of Nrf2 target genes compared with K5cre-CMVcaNrf2 mice (Schäfer *et al*, 2010). K5cre-caNrf2 mice also had curly hairs and they lost pelage hairs, but this was only observed 5–6 weeks after birth and was restricted to the tail (Supplementary Fig 4A–C). This locally restricted phenotype correlates with the stronger expression of *Nrf2* (endogenous *Nrf2* plus transgene-derived *caNrf2*) and its target genes *Nqo1*, *Gclc, Gclm,* and sulfiredoxin1 (*Srxn1*) in the tail compared with the back skin (Supplementary Fig 4D). Histological analyses and Adph immunofluorescence staining of tail skin sections identified enlarged SGs, an increased number of sebocytes (Supplementary Fig 4E–H), sebum-filled infundibula (Supplementary Fig 4E arrow), and dilatation of infundibula (Supplementary Fig 4F). These abnormalities were similar as in K5cre-CMVcaNrf2 mice, but less pronounced, demonstrating a strong dependency of the phenotype on the level of Nrf2 activation.

### Aged K5cre-CMVcaNrf2 mice develop large cutaneous and meibomian cysts

In one-year-old (1 y) K5cre-CMVcaNrf2 mice, we found a more severe dilatation and keratinization of infundibula in back (Supplementary Fig 5A) and tail skin (Fig 2C) when compared to young mice. In addition, keratinized infundibular cysts of different sizes (Fig 2C) and a few macroscopically visible large cysts were observed in the tail skin (Fig 2D, Supplementary Table 1). The cutaneous cysts were mainly filled with keratin and to a much lesser extent with lipids as indicated by H&E and oil red O staining (Fig 2C,E). In addition, serial sections revealed that only small or no SGs are associated with the large cysts. In some of the large cysts, we found a rupture of the epithelial wall, most likely due to keratin overproduction and consequent enlargement of the cyst lumen (Supplementary Fig 5B). These cysts are of hair follicle origin, since the epithelial cells expressed K14 (ORS-and basal infundibulum cells), keratin K6 (ORS-and active/hyperproliferative infundibulum cells), but not K10 and loricrin (Lor) (both missing in ORS cells) (Supplementary Fig 5C). Thus, in aged K5cre-CMVcaNrf2 mice, the continuous hyperkeratinization of infundibula, which starts already in young mice, leads to progressive dilatation of infundibula and ultimately to formation of cutaneous cysts (Fig 2F).

In preputial glands of aged K5cre-CMVcaNrf2 mice, where caNrf2 is also strongly expressed as reflected by the strong expression of its target genes (Supplementary Fig 5D), we observed hyperkeratosis of the luminal duct (Supplementary Fig 5E). Ultimately, this resulted in preputial gland swelling due to sebum congestion (Supplementary Fig 5F,G).

### Activation of Nrf2 induces sebocyte hyperproliferation and disturbs sebocyte differentiation and lipid metabolism

**Figure 3:**
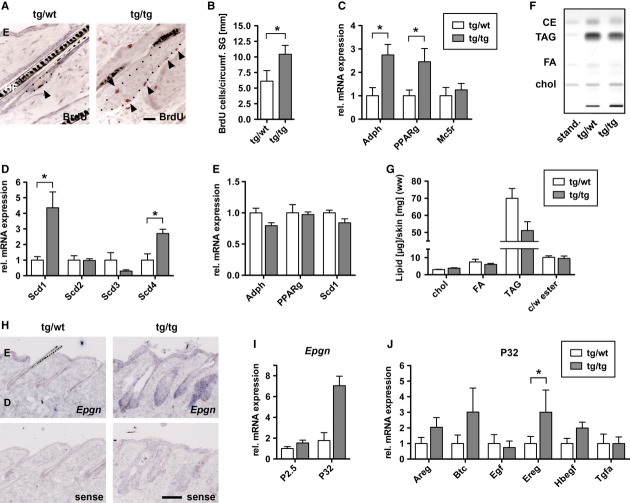
Sebocyte proliferation, differentiation, and lipid metabolism in K5cre-CMVcaNrf2 mice. A BrdU staining on skin sections of tg/wt and tg/tg mice. Arrowheads point to BrdU-positive sebocytes. The dotted line marks the boundary of the SGs. Scale bar: 50 μm. B Quantification of BrdU-positive sebocytes per circumference of SG in P32 tg/wt and tg/tg mice (*N* = 4, *P = 0.0286). C, D qRT-PCR for *Adph* (*N* = 4, *P = 0.0286), *Pparg* (*N* = 4, *P = 0.0286) and *Mc5r* (*N* = 3) (C) and *Scd1* (*P = 0.0286), *Scd2*, *Scd3,* and *Scd4* (*P = 0.0286, *N* = 4) (D) relative to *Gapdh* using RNAs from skin of P32 control (tg/wt) and K5cre-CMVcaNrf2 (tg/tg) mice. Expression in tg/wt mice was arbitrarily set as 1 (dashed line). E qRT-PCR of *Adph*, *Pparg* and *Scd1* in P2.5 control and K5cre-CMVcaNrf2 mice (*N* = 3). F Separation of cholesterol (Chol), free fatty acids (FA), triacylglycerides (TAG), and cholesterol-and wax esters (CE) by HPTLC in control (tg/wt) and K5cre-CMVcaNrf2 (tg/tg) mice. G Wet weight ratio of cholesterol, free fatty acids, triacylglycerides, and cholesterol-and wax esters relative to total skin protein. Note the reduction in TAG levels in tg/tg mice. H *In situ* hybridization on back skin sections from 8-w-old tg/wt and tg/tg mice using *Epgn* sense (upper panel) and antisense probes (lower panel). Note strong staining in interfollicular epidermis and pilosebaceous unit, particularly on tg/tg section hybridized with the *Epgn* antisense probe. Scale bar: 50 μm. I qRT-PCR of *Epgn* relative to *Gapdh* using RNAs from skin of P2.5 and P32 tg/wt and tg/tg mice (*N* = 3). Expression in tg/wt mice was arbitrarily set as 1 (dashed line). J qRT-PCR of epidermal growth factor (EGF) family members in P32 tg/wt and tg/tg mice (*N* = 3/4). Data information: Values are shown as the mean with s.d. All *P*-values were calculated by Mann–Whitney *U*-test.

In the back of P32 K5cre-CMVcaNrf2 mice as well as in the tail of K5cre-caNrf2 mice, we found a significant increase in the number of proliferating sebocytes in relation to the circumference of the SG as determined by 5-bromo-2`-deoxyuridine (BrdU) labeling (Fig 3A,B and Supplementary Fig 6A,B). This provides a likely explanation for the severe SG hyperplasia in these mice. Furthermore, expression of the SG markers *Adph* and peroxisome proliferator-activated receptor gamma (*Pparg*) (Rosenfield *et al*, 1999) (Fig 3C) was increased, which correlates with the enlargement of SG in these mice. However, the late SG differentiation marker melanocortin-5 receptor (*Mc5r*) (Chen *et al*, 1997; van der Kraan *et al*, 1998) was not upregulated concomitantly (Fig 3C), indicating a disturbance in late differentiation or maturation of sebocytes. Consistent with this observation, we found a differential expression of stearoyl-CoA desaturases (Scds; D^9^ desaturases), which catalyze the conversion of saturated into mono-unsaturated fatty acids (Guillou *et al*, 2010). *Scd1* was strongly upregulated, while *Scd3* was downregulated in the skin of K5cre-CMVcaNrf2 mice (Fig 3D). Similar alterations in the expression of these genes were also detected in wild-type mice upon activation of endogenous Nrf2 by repeated tBHQ treatment (Supplementary Fig 6D,E). However, expression of *Adph, Pparg,* and *Scd1* were not significantly upregulated at P2.5 in K5cre-CMVcaNrf2 mice (Fig 3E), suggesting that the regulation of these genes is not a direct consequence of Nrf2 activation, but rather a consequence of the increase in sebocyte proliferation and abnormalities in sebocyte differentiation.

High-performance thin-layer chromatography of the back skin lipids of K5cre-CMVcaNrf2 mice revealed a 27% reduction in triacylglycerides, while the levels of fatty acids, cholesterol, and cholesterol-and wax esters were normal (Fig 3F,G). These alterations in sebocyte lipid metabolism may involve the deregulation of *Scds* in K5cre-CMVcaNrf2 mice.

### Nrf2 activation induces hyperproliferation of sebocytes by upregulation of epigen

To determine the molecular mechanisms underlying the sebocyte phenotype, we analyzed microarray data from skin of P2.5 control and K5cre-CMVcaNrf2 mice (Schäfer *et al*, 2012). Interestingly, this analysis revealed upregulation of the epidermal growth factor (EGF) family member epigen (Epgn), which had previously been shown to induce sebocyte enlargement when overexpressed in transgenic mice (Dahlhoff *et al*, 2009). *In situ* hybridization revealed strong *Epgn* expression in the interfollicular epidermis and the pilosebaceous unit, including SGs, in control and in particular in K5cre-CMVcaNrf2 mice (Fig 3H, Supplementary Fig 6F representing different staining intensities). Furthermore, qRT-PCR analysis confirmed upregulation of *Epgn* in the back skin of P2.5 and P32 K5cre-CMVcaNrf2 mice (Fig 3I) and in their preputial glands (Supplementary Fig 6G), as well as in the tail skin of K5cre-caNrf2 mice (Supplementary Fig 6C). We also found increased expression of other EGF family members, including amphiregulin (*Areg*), betacellulin (*Btc*), epiregulin (*Ereg*), and heparin-binding EGF-like growth factor (*Hbegf*) in the skin of P32 (Fig 3J), but not of P2.5 K5cre-CMVcaNrf2 mice.

**Figure 4:**
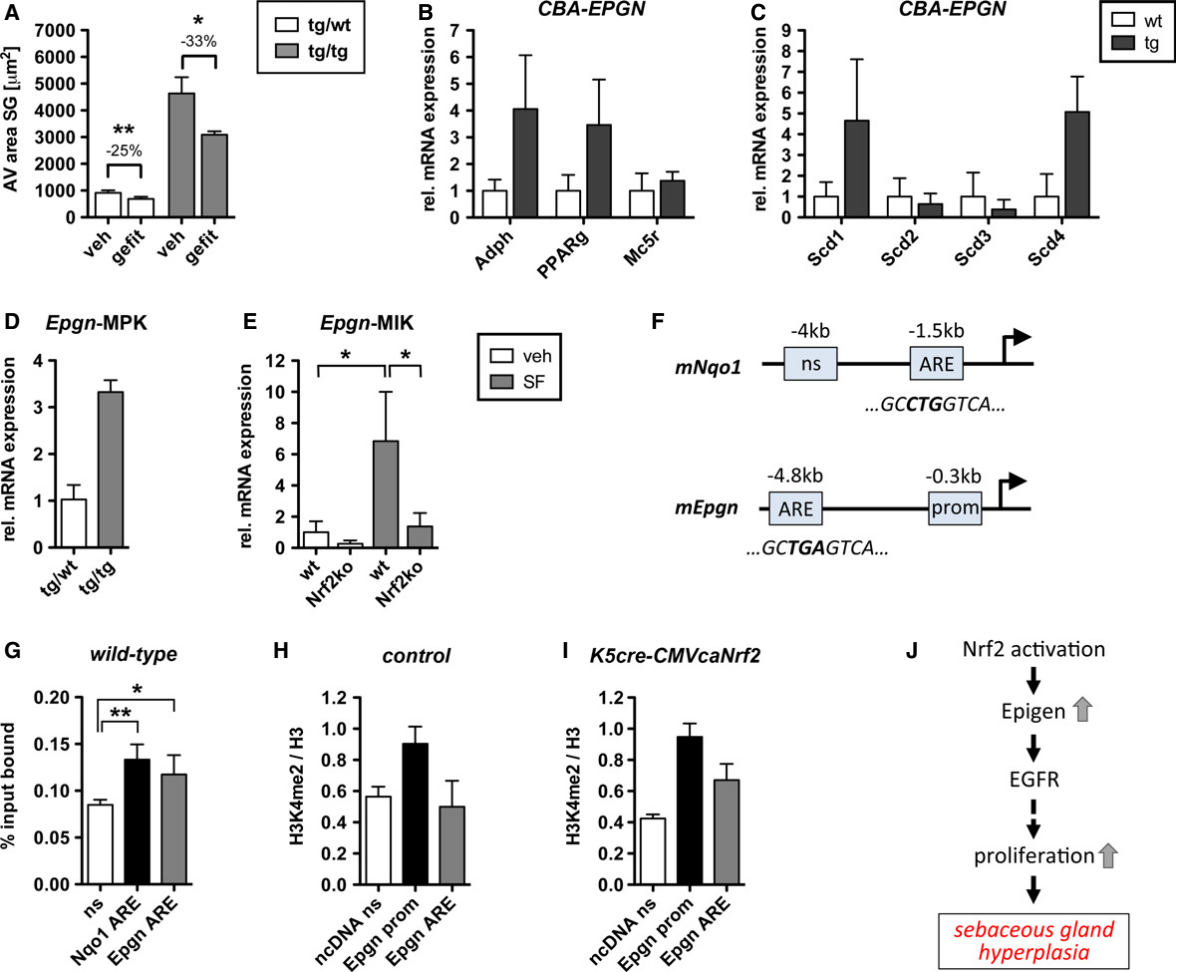
Regulation of Epgn by Nrf2. A Average SG area of P32 tg/wt and tg/tg mice injected with vehicle or Gefitinib. Note reduction in SG area upon Gefitinib treatment of tg/wt mice (*N* = 6/5, **P = 0.0025) and tg/tg mice (*N* = 4, *P = 0.0286). B, C qRT-PCR of *Adph*, *Pparg* *Mc5r* (B), and *Scd1*, *2*, *3*, *4* (C) relative to *Gapdh* using RNA of back skin from wt and *EPGN*-tg mice (*N* = 3). D, E qRT-PCR of *Epgn* using RNAs of murine primary keratinocytes of tg/wt and tg/tg mice (*N* = 3) (D), or immortalized keratinocytes of wt and *Nrf2ko* mice treated with vehicle or sulforaphane (E) (*N* = 4, *P = 0.0286). F Localization of PCR-amplified non-specific region (ns), promoter region (prom), and antioxidant response elements (AREs) in the murine *Nqo1* and *Epgn* genes. G Chromatin immunoprecipitation (ChIP) using lysates from back and tail skin of control mice. Note the enhanced binding of Nrf2 to the *Epgn* ARE compared with the ns region (*Nqo1* ns, *N* = 6, *Nqo1* ARE, *N* = 6, **P = 0.0045; *Epgn* ARE, *N* = 4, *P = 0.012). H, I ChIP using back and tail skin lysates of tg/wt (H) and tg/tg mice (I) with an H3K4me2 antibody. Regions void of annotated ORFs (non-coding, nc DNA), *Epgn* prom, and *Epgn* ARE were amplified by qRT-PCR. Note the increase in histone H3 dimethylation at the *Epgn* ARE compared with *ncDNA* in tg/tg mice (*N* = 2/3). J Working model: Nrf2 activation leads to increased *Epgn* expression, which activates EGFR signaling. This enhances proliferation of sebocyte progenitors, leading to sebaceous gland hyperplasia. Data information: Values are shown as the mean with s.d. All *P*-values were calculated by Mann–Whitney *U*-test.

Inhibition of EGFR signaling by systemic treatment of control and K5cre-CMVcaNrf2 mice with the EGFR kinase inhibitor Gefitinib (Iressa) resulted in a 25% reduction in SG area of control mice compared with vehicle-treated mice, and an even stronger reduction (33%) in K5cre-CMVcaNrf2 mice (Fig 4A). These results suggest that the hyperproliferation and hyperplasia of the SG are the consequence of elevated expression of EGF family members, followed by enhanced EGFR signaling. Epgn seems to be the initial growth stimulus, since it was upregulated in P2.5 K5cre-CMVcaNrf2 mice and thus prior to the development of the SG phenotype. Consistent with this hypothesis, transgenic mice expressing human *EPGN* under control of a CMV enhancer and β-actin promoter also showed enhanced sebocyte proliferation and severe SG hyperplasia (Dahlhoff *et al*, 2009). Moreover, the regulation of *Adph, Pparg Mc5r,* and *Scd1*, *2*, *3,* and *4* was similar in *EPGN* transgenic mice and K5cre-CMVcaNrf2 mice (Fig 4B,C; see Fig 3C,D).

### Epigen is a direct target of Nrf2, which is upregulated upon Nrf2 activation

Upregulation of *Epgn* expression was observed in primary keratinocytes from K5cre-CMVcaNrf2 mice (Fig 4D) as well as in sulforaphane-treated keratinocytes from wild-type mice, but not from *Nrf2ko* mice (Fig 4E). Thus, it occurs in a cell-autonomous and Nrf2-dependent manner, suggesting that *Epgn* is a direct Nrf2 target gene. This hypothesis is supported by *in silico* identification of a classical antioxidant response element (ARE; Nrf2 binding site) (Rushmore *et al*, 1991) in the murine *Epgn* gene promoter, 4.8 kb upstream of the transcription start site (Fig 4F). Chromatin immunoprecipitation (ChIP) using a mixture of lysates from back and tail epidermis of wild-type mice using an Nrf2 antibody revealed binding of endogenous Nrf2 to the *Epgn* ARE, but not to a different promoter region (Fig 4G). ChIP analysis of lysates using a histone H3 dimethyl Lys4 (H3K4me2) antibody, a marker of transcriptionally active chromatin (Pekowska *et al*, 2011), showed no increase in histone H3 dimethylation at the *Epgn* ARE compared with a non-coding DNA region (ncDNA) in control mice (Fig 4H). This suggests that *Epgn* gene expression is not activated by endogenous Nrf2 under homeostatic conditions. However, there was an increase in histone H3 dimethylation at the *Epgn* ARE in K5cre-CMVcaNrf2 mice (Fig 4I), indicating that caNrf2 activates *Epgn* transcription via binding to the identified ARE. Taken together, these data reveal that Nrf2 binds to the *Epgn* ARE and that Nrf2 activation enhances *Epgn* gene transcription. This leads to increased EGFR signaling and concomitant induction of sebocyte proliferation, resulting in sebaceous gland hyperplasia (Fig 4J).

### Acanthosis and hyperkeratosis of infundibula in K5cre-CMVcaNrf2 mice are most likely the consequence of Slpi, Sprr2d, and Epgn upregulation

**Figure 5:**
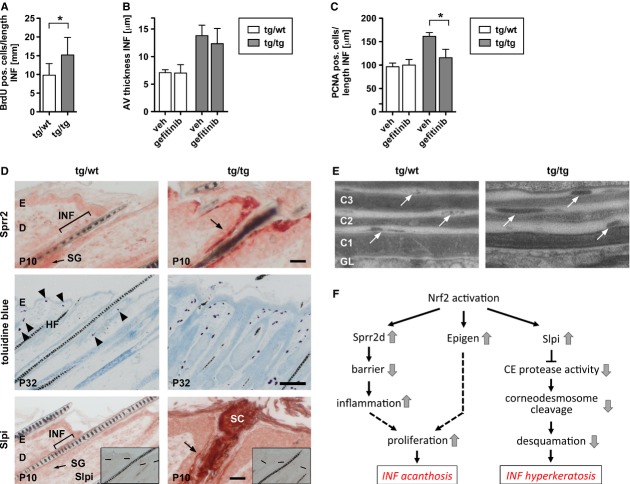
Sprr2d and Slpi expression in infundibula of K5cre-CMVcaNrf2 mice. A Average number of BrdU-positive cells in infundibula (INF) of tg/wt and tg/tg mice (*N* = 4). B,C Average thickness of infundibula and number of PCNA-positive cells in infundibula of P32 tg/wt and tg/tg mice injected with vehicle or Gefitinib (*N* = 4/5). D Upper panel: immunohistochemistry of Sprr2 on longitudinal P10 tg/wt and tg/tg back skin sections. Note staining of Sprr2 in differentiated infundibular keratinocytes in tg/tg mice. Scale bar: 25 μm. Middle panel: toluidine blue staining for detection of mast cells on longitudinal back skin sections of P32 tg/wt and tg/tg mice. Note increase in number of mast cells in tg/tg mice and assembly of mast cells along the interfollicular epidermis and upper part of the hair follicles. Scale bar: 100 μm. Lower panel: immunohistochemistry of Slpi on longitudinal P10 tg/wt and tg/tg back skin sections. Inset in lower panel shows immunohistochemistry without primary antibody. The dashed line marks the basement membrane of the interfollicular epidermis. Note staining of Slpi (indicated by arrow) in differentiated infundibular keratinocytes and *stratum corneum* in tg/tg mice. Scale bar: 25 μm. E  Electron microscopy of infundibular *stratum corneum* of P32 tg/wt and tg/tg mice. Corneocyte layers in lower panel were numbered from basal to distal (C1–C3). Arrows point to corneodesmosomes. Note delayed corneodesmosome degradation in tg/tg mice. C, corneocyte. F Working model: Nrf2 activation leads to upregulation of Slpi, Sprr2d, and Epgn in hair follicle infundibula. Slpi upregulation promotes inhibition of CE protease activity. Consequently, corneodesmosome cleavage is reduced, leading to decreased desquamation and thereby to hyperkeratosis of infundibula. Sprr2d upregulation reduces epidermal barrier functionality, resulting in increased inflammation and consequently stimulation of proliferation. Epgn and other epidermal growth factor (EGF) family members stimulate proliferation of infundibular keratinocytes via EGFR signaling. Together, this results in acanthosis of hair follicle infundibula. Data information: Values are shown as the mean with s.d.

BrdU labeling showed that thickening of hair follicle infundibula results from increased proliferation of keratinocytes (Fig 5A). Gefitinib treatment caused a minor reduction in the thickness of infundibula (Fig 5B), but a significant reduction in the proliferation rate of keratinocytes compared with vehicle-treated mice (Fig 5C). Thus, activation of EGFR signaling obviously enhances keratinocyte proliferation and hyperplasia of infundibula in K5cre-CMVcaNrf2 mice.

In the epidermis, Nrf2-mediated upregulation of Sprr2d reduced the mechanical stability of the corneocytes, resulting in an impairment of epidermal barrier function (Schäfer *et al*, 2012). This deficiency caused mild inflammation and upregulation of keratinocyte mitogens, resulting in keratinocyte hyperproliferation. We also found upregulation of Sprr2 expression in differentiated keratinocytes of infundibula (Fig 5D upper panel) and a clustering of mast cells around the hair follicles of K5cre-CMVcaNrf2 mice (Fig 5D middle panel). This result strongly suggests that Sprr2d upregulation causes acanthosis of infundibula by reducing barrier functionality (see model in Fig 5F).

Slpi, which inhibits kallikrein 7, a protease required for corneodesmosome cleavage (Franzke *et al*, 1996), was significantly upreg-ulated in the epidermis of K5cre-CMVcaNrf2 transgenic mice, leading to reduced desquamation and consequently hyperkeratosis (Schäfer *et al*, 2012). We observed a significant overexpression of Slpi in the IFN and infundibular *stratum corneum* (Fig 5D lower panel) and impaired corneodesmosome degradation in the IFN (Fig 5E). Thus, follicular hyperkeratosis in K5cre-CMVcaNrf2 mice most likely results from increased expression of Slpi, which inhibits corneocyte desquamation (Fig 5F).

Interestingly, Sprr2d and Slpi were both upregulated in preputial glands of K5cre-CMVcaNrf2 mice, which became hyperkeratotic upon aging (Supporting Information Fig 6G), suggesting a similar mechanism of action of activated Nrf2 in skin and preputial glands.

**Figure 6:**
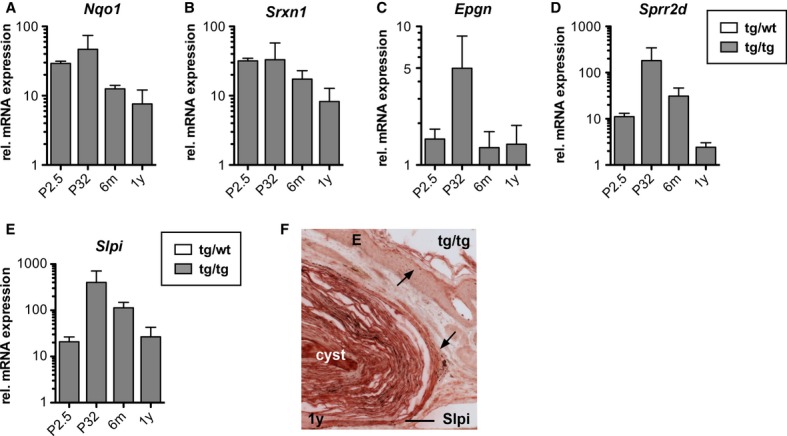
Differential regulation of Nrf2 target genes in K5cre-CMVcaNrf2 mice. A–E qRT-PCR for *Nqo1*, *Srxn1*, *Epgn*, *Sprr2d,* and *Slpi* relative to *Gapdh* using RNAs from skin of P2.5, P32, 6-m-and 1-y-old tg/wt and tg/tg mice (*N* = 3/4). Expression in tg/wt mice was arbitrarily set as 1. F Immunohistochemistry of Slpi on 1 y tg/wt and tg/tg back skin sections. Note strong staining of Slpi in cyst epithelium and keratinized lumen compared with differentiated keratinocytes of the epidermis (indicated by arrow). Scale bar: 100 μm. Data information: Values are shown as the mean with s.d.

### Cyst formation in K5cre-CMVcaNrf2 mice is the consequence of continuous follicular hyperkeratosis

K5cre-CMVcaNrf2 mice had a milder epidermal phenotype at 6 m compared with P32 due to partial silencing of *caNrf2* expression and consequent downregulation of Nrf2 target genes (Schäfer *et al*, 2012). At the age of 1 year, they showed an even further decrease in expression of the classical Nrf2 target genes *Nqo1* (Fig 6A) and *Srxn1* (Fig 6B). Expression of *Epgn* had significantly declined at 6 m (Fig 6C), providing a likely explanation for the low sebum content in the cyst lumen (see Fig 2E) and the reduced size or even absence of SGs associated with large cysts. The decline in *Sprr2d* (Fig 6D) and *Slpi* (Fig 6E) expression upon aging of K5cre-CMVcaNrf2 mice, however, was less pronounced, and in particular *Slpi* mRNA levels were still more than 20-fold higher than in control animals at the age of 1 year (Fig 6E). Consistently, immunohistochemistry revealed strong staining for Slpi in the epithelium and keratin-filled lumen of the cysts of aged K5cre-CMVcaNrf2 mice (Fig 6F). Thus, continuous high expression of Slpi and a consequent reduction of corneocyte desquamation seem to underlie the development of keratinized cysts in aged mice. These findings suggest that differences in the ratio of individual Nrf2 targets result in different cutaneous abnormalities.

### SPRR2 and SLPI are strongly expressed in cysts of MADISH patients

**Figure 7:**
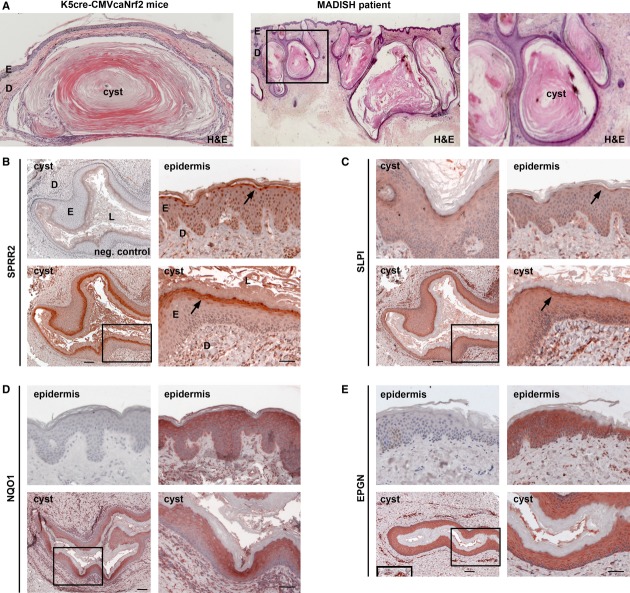
Histopathological features and expression of SPRR2D and SLPI in MADISH patients. A Left: transverse section of tail skin from 1-y-old K5cre-CMVcaNrf2 mouse with large cyst. Middle: section of pre-auricular skin biopsy of a MADISH patient. Right: close-up as indicated by rectangle in middle picture. Note lack of SGs and large cutaneous keratinized cysts in skin section of K5cre-CMVcaNrf2 mouse and MADISH patient. B, C Immunohistochemistry staining of SPRR2 (B) and SLPI (C) on skin section of MADISH patients. Left upper picture in (B): negative control lacking primary antibody and with secondary antibody used for SPRR2 and SLPI staining. Left upper picture in (C): SLPI staining in cyst epithelium. Right upper picture: SPRR2 (B) and SLPI staining (C) in epidermis (arrow). Lower panel: cyst in overview (left) and close-up as indicated by rectangle. Note strong SPRR2 and SLPI staining in differentiated cyst keratinocytes and epidermis (arrow). Scale bars: 100 μm (left), 50 μm (right). D, E Immunohistochemistry of NQO1 and EPGN on skin section of MADISH patient. Upper left picture: negative control secondary antibody only. Upper right picture: NQO1 (D) and EPGN staining (E) in epidermis. Lower panel: cyst in overview (left) and close-up as indicated by rectangle. Note strong NQO1 staining in differentiated keratinocytes and strong EPGN staining in all keratinocytes of cysts and epidermis (arrow). Inset in lower left panel in (E) shows EPGN staining in endothelial cells (positive control). Scale bars: 100 μm (left) and 50 μm (right).

The histopathological features of K5cre-CMVcaNrf2 mice showed remarkable similarities to the cutaneous abnormalities seen in patients with chloracne/MADISH (Saurat & Sorg, 2010), including hair follicle proliferation, hyperplasia, and hyperkeratosis as well as formation of keratinized cysts with small or no SGs (Fig 7A) (Panteleyev & Bickers, 2006). This prompted us to investigate the potential involvement of similar pathomechanisms in caNrf2-transgenic mice and MADISH patients. Indeed, we detected strong expression of SPRR2, SLPI, EPGN and of the classical NRF2 target NQO1 in the epidermis and cyst epithelium of MADISH patients, with particularly strong staining of SPRR2, SLPI, and NQO1 in differentiated keratinocytes (Fig 7B–E). Since all these genes are concomitantly activated by caNrf2 in mouse keratinocytes *in vivo*, it seems most likely that NRF2 is activated in keratinocytes of MADISH patients. The consequent upregulation of SLPI, SPRR2, and EPGN could then contribute to cyst formation in the skin of MADISH patients by the observed interference with stratum corneum desquamation and epidermal barrier formation.

### Dioxin treatment enhances expression of SLPI, SPRR2D, and EPGN in human keratinocytes in an Nrf2-dependent manner

**Figure 8:**
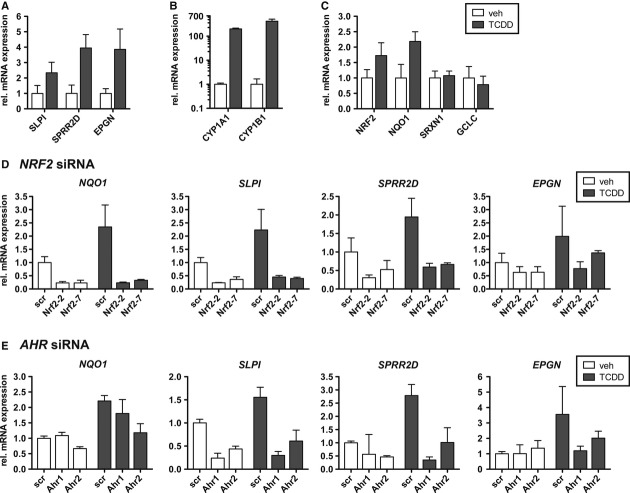
TCDD upregulates *SLPI*, SPRR2D, and EPGN in an AHR-and NRF2-dependent manner in human foreskin keratinocytes. A–C qRT-PCR for *SLPI, SPRR2D,* and *EPGN* (A), *CYP1A1* and *CYP1B1* (B), *NRF2*, *NQO1, SRXN1*, and *GCLC* (C), relative to *GAPDH* using RNAs from 10^−8^ M TCDD and vehicle-treated human foreskin keratinocytes (HFKs) (*N* = 3). Expression in vehicle-treated HFKs was arbitrarily set as 1. D, E qRT-PCR of *NQO1*, *SLPI, SPRR2D,* and *EPGN* relative to *GAPDH* using RNA from HFKs transfected with siRNA targeting *NRF2* (Nrf2-2 or Nrf2-7) (D) or AHR (AHR1 or AHR2) (E) or scrambled (scr) siRNA (D,E) (*N* = 3/4). Cells were treated with 10^−8^ M TCDD or vehicle. Note downregulation of *NQO1*, *SLPI, SPRR2D,* and *EPGN* in cells transfected with siRNAs targeting *NRF2* or *AHR*. Data information: Values are shown as the mean with s.d.

The pathology of MADISH patients develops after intoxication with halogenated aromatic hydrocarbons, such as 2,3,7,8-tetrachlorodibenzo-p-dioxin (TCDD) (Geusau *et al*, 2001; Sorg *et al*, 2009; Saurat *et al*, 2012). Interestingly, we found upregulation of *SLPI*, *SPRR2D,* and *EPGN* in cultured human foreskin keratinocytes (HFKs) by treatment with TCDD (Fig 8A). TCDD is known to activate the aryl hydrocarbon receptor (AHR), and this was verified by the strong increase in the expression of cytochrome P450 1A1 and 1B1 (*CYP1A1* and *CYP1B1)* (Fig 8B), which are major AHR target genes (Hayes *et al*, 2009). Concomitantly, expression of *NRF2* and *NQO1* was induced (Fig 8C). This finding is consistent with previous observations showing that TCDD via AHR leads to increased expression and/or activation of NRF2 in different cell types (Hayes *et al*, 2009). We next transfected HFKs with two different siRNAs targeting *NRF2* mRNA (Supplementary Fig 7A). Interestingly, the basal and TCDD-induced expression of *NQO1*, *SLPI*, *SPRR2D,* and *EPGN* was strongly diminished after transfection with *NRF2* siRNA (Fig 8D). Furthermore, transfection with two different siRNAs targeting *AHR* (Supplementary Fig 7B) resulted in a strong reduction of the TCDD-induced expression of *SLPI*, *SPRR2D,* and *EPGN* (Fig 8E). These data provide evidence for a TCDD-induced activation of an AHR-NRF2 axis in MADISH patients, which results in upregulation of the NRF2 targets *SLPI*, *SPRR2D,* and *EPGN* with subsequent development of cystic lesions.

## Discussion

Nrf2-activating compounds are used for skin protection under stress conditions as well as for cancer prevention. Unfortunately, there is accumulating evidence for severe adverse effects of Nrf2 activation. In this study, we identified unexpected abnormalities in the pilosebaceous unit upon long-term activation of Nrf2 in the skin. The phenotype was dependent on the level and duration of Nrf2 activation, which resulted in different ratios of Nrf2 targets. This needs to be considered when Nrf2-activating compounds are used for skin protection *in vivo*.

We identified acanthosis and hyperkeratosis of infundibula and the interfollicular epidermis as the reason for dilatation of infundibula in caNrf2-transgenic mice. These alterations prevent sebum outflow due to narrowing and plugging of the hair canal. An increase in sebum production by the enlarged SG further aggravates sebum congestion. The dilatation of infundibula, in turn, reduces the anchorage of hairs, providing a likely explanation for their premature loss during late catagen and telogen. By contrast, we excluded alterations in hair follicle morphogenesis or cycling or in the differentiation of hair follicle keratinocytes. The follicular and epidermal hyperkeratosis and acanthosis also explain the thinning and malformation of hairs, because they prevent a proper outgrowth of newly forming hairs during anagen. The hair loss also promotes the occlusion of the hair canal and thereby sebum congestion and dilatation of infundibula.

Several results of this study strongly suggest that Nrf2-induced upregulation of *Epgn* is the primary reason for the observed SG abnormalities and the acanthosis of infundibula: (i) *Epgn* was upreg-ulated in K5cre-CMVcaNrf2 mice prior to the development of histological abnormalities, and it was strongly expressed in the pilosebaceous unit, including the SGs of adult K5cre-CMVcaNrf2 mice; (ii) *EPGN* transgenic mice showed similar abnormalities of the pilosebaceous unit and epidermis (Dahlhoff *et al*, 2009), including a similar expression profile of genes involved in sebocyte differentiation and lipid metabolism (this study); (iii) pharmacological blockade of EGFR signaling reduced hyperplasia of SGs and proliferation of infundibula in K5cre-CMVcaNrf2 mice; (iv) *Epgn* upregulation occurred in a cell-autonomous and Nrf2-dependent manner in cultured keratinocytes; and (v) ChIP experiments revealed that Nrf2 binds to a distal ARE in the *Epgn* promoter region and that Nrf2 activation causes transcriptional activation of *Epgn*.

In addition to this direct effect, acanthosis of infundibula in K5cre-CMVcaNrf2 mice may result at least in part from upregulation of Sprr2d and subsequent disruption of the follicular barrier. This allows the penetration of allergens and/or bacteria from the lumen of hair follicles, which are colonized by commensal microbes (Nakatsuji *et al*, 2013). The resulting accumulation of immune cells stimulates proliferation of keratinocytes in a paracrine manner through upregulation of keratinocyte mitogens as previously demonstrated for epidermal keratinocytes (Schäfer *et al*, 2012). The infundibular hyperkeratosis and impaired *stratum corneum* desquamation can be explained by overexpression of *Slpi*, a direct target of Nrf2 (Iizuka *et al*, 2005), in the infundibula of K5cre-CMVcaNrf2 mice. The previously demonstrated inhibition of kallikrein 7 activity by Slpi (Franzke *et al*, 1996) likely inhibits corneodesmosome cleavage in the infundibular *stratum corneum*, resulting in impaired desquamation and ultimately follicular hyperkeratosis. Taken together, these results reveal previously unrecognized functions of Slpi, Sprr2d, and Epgn in the pilosebaceous unit.

In aged K5cre-CMVcaNrf2 mice, we observed large keratin-filled cysts, in particular in the tail. The different histopathological abnormalities in aged versus young mice may be due to different ratios in the expression levels of individual Nrf2 target genes. In general, there was an age-dependent decline in the expression of Nrf2 target genes in K5cre-CMVcaNrf2 mice. *Epgn* expression showed a peak at P32, but was strongly reduced already in 6-m-old mice. This provides a likely explanation for the marginal sebum in the cyst lumen and the reduced size or lack of SG associated with cysts in 1-year-old K5cre-CMVcaNrf2 mice. By contrast, the age-dependent decline in *Sprr2d* and in particular in *Slpi* expression was less pronounced. The continuously high expression of these proteins is likely to be responsible for the progressive hyperkeratinization of infundibula and formation of keratinized cutaneous cysts upon aging.

Follicular hyperkeratosis and acanthosis together with SG hyperplasia and seborrhea as seen in young K5cre-CMVcaNrf2 mice are important characteristics of acne in humans. However, the development of *Acne Vulgaris* also involves *Propionibacterium (P.) acnes* overgrowth, which only affects humans, but not mice, and which triggers a severe inflammatory response (Makrantonaki *et al*, 2011). K5cre-CMVcaNrf2 transgenic mice only had mild inflammation, presumably due to an epidermal barrier defect (Schäfer *et al*, 2012), but not as a result of *P. acnes* overgrowth. Therefore, the phenotype of caNrf2 mice rather resembles the abnormalities seen in human non-inflammatory acne, in particular *Acne Comedonica*.

The keratinization of the cyst lumen and reduction or lack of SGs in aged mice, in contrast, are remarkably reminiscent to the pathology that develops in MADISH patients (Panteleyev & Bickers, 2006; Saurat *et al*, 2012). Indeed, we found strong expression of NQO1, SLPI, SPRR2, and EPGN in the affected epidermis and cyst epithelium of these patients. The nuclear staining of SPRR2 in the human epidermis is consistent with the nuclear localization and DNA binding activity of SPRR2 that protects DNA from ROS damage (Vermeij *et al*, 2012). Upregulation of *Slpi*, *Sprr2d,* and *Epgn* was detected by microarray analysis using RNA of P2.5 K5cre-CMVcaNrf2 mice (Schäfer *et al*, 2012). These genes seem to be the major drivers of the skin phenotype, since no other genes encoding growth factors or proteins involved in epidermal barrier function/keratinization were regulated at this early time point. It had previously been shown that TCDD induced the expression of various genes of the human epidermal differentiation complex (Sutter *et al*, 2011). However, we did not detect an increase in the expression of these genes in P2.5 K5cre-CMVcaNrf2 mice by microarray analysis, and this was confirmed for loricrin (Schäfer *et al*, 2012), *Sprr1a* (Schäfer *et al*, 2012), *Sprr2a*, late cornified envelope (*Lce*)*1a*, and *S100A7* by qRT-PCR (Supplementary Fig 6H), indicating that these genes do not contribute to the phenotype. Rather, the combination of SPRR2D and the newly identified TCDD targets *SLPI* and *EPGN* is most likely sufficient to induce the MADISH-like pathology in K5cre-CMVcaNrf2 mice and also in MADISH patients. Thus, the mechanistic studies performed in mice together with the strong expression of SLPI, SPRR2, and EPGN in affected skin of MADISH patients provide strong evidence for a contribution of these proteins to the cyst development in MADISH patients by mediating follicular hyperplasia and hyperkeratosis.

Metabolizing acquired dioxin-induced skin hamartomas develops upon exposure to halogenated aromatic hydrocarbons, such as TCDD, which activates AHR. The important role of AHR in MADISH pathogenesis is supported by the finding that transgenic mice expressing a constitutively active mutant of the Ahr receptor (caAhr) in keratinocytes also develop epidermal cysts (Tauchi *et al*, 2005). It has previously been shown that TCDD-activated AHR induced NRF2 activation in different cell types *in vitro* (Hayes *et al*, 2009), and Nrf2 was also activated in transgenic mice expressing caAhr in keratinocytes (Tauchi *et al*, 2005). However, a role of NRF2 in TCCD-induced cyst development in MADISH patients has not been demonstrated so far. We found that TCCD stimulated the expression of the classical NRF2 target *NQO1* in human foreskin keratinocytes, and TCCD-mediated upregulation of *SLPI*, *SPRR2D,* and *SLPI* was NRF2 and AHR dependent. Furthermore, NQO1 was strongly expressed in the cyst epithelium of MADISH patients. This suggests that *SLPI*, *SPRR2D,* and *SLPI* are also upregulated by TCDD-mediated activation of AHR and NRF2 *in vivo* under these conditions. By contrast, upregulation of these genes is not a general response of hyperproliferative keratinocytes, since it was not observed in human basal cell carcinomas (Supplementary Fig 7C).

Although TCCD induced the expression of *NQO1* and the novel NRF2 targets *EPGN*, *SPRR2D,* and *SLPI* in cultured human keratinocytes in an NRF2-dependent manner, no significant upregulation of various other classical NRF2 target genes was detected. It may well be that the rather weak activation of NRF2 by TCCD is insufficient to induce the expression of most classical NRF2 target genes, with the exception of *NQO1*, which is particularly sensitive to NRF2 activation. TCDD-mediated induction of *SPRR2D*, *SLPI,* and *EPGN* may be achieved by a combination of NRF2 and AHR that is more potent than NRF2-mediated activation alone. This novel NRF2-AHR cross-talk needs further investigations at the molecular level. Independent of the regulatory mechanisms, our data provide strong evidence for a potent function of the newly discovered AHR-NRF2-SLPI/SPRR2D/EPGN axis in the pathogenesis of MADISH. Since this axis is also activated in K5cre-CMVcaNrf2 mice, these animals provide an interesting novel model to study this important toxin-induced disorder.

## Materials and Methods

### RNA isolation and qRT-PCR

Total cellular RNA was isolated as previously described (Chomczynski & Sacchi, 1987; Werner *et al*, 1993) and further purified using the Nucleospin RNAII kit (Macherey-Nagel, Düren, Germany) or the MinElute kit (Qiagen, Hilden, Germany). cDNA synthesis and qRT-PCR were described previously (Yang *et al*, 2010). Primers used are listed in Supplementary Table 2.

### Histological and immunohistological stainings and labeling with BrdU

Histological and immunofluorescence analyses were described earlier (Schäfer *et al*, 2010). For antigen retrieval, PFA-fixed paraffin sections were heated up from room temperature to 95°C and incubated for 1 h at 95°C (for SLPI, SPRR2D, and EPGN). Primary antibodies used were anti-Adph (Fitzgerald, Acton, MA, USA), anti-BrdU-POD (Roche, Rotkreuz, Switzerland), anti-PCNA, and anti-Slpi (all from Santa Cruz, CA, USA), anti-Nrf2 (Huebner *et al*, 2012), anti-H3K4me2 (Active Motif, Carlsbad, CA, USA), anti-Epgn, anti-NQO1 (Abcam, Cambridge, UK), anti-SPRR2 (kindly provided by Dr. Daniel Hohl, Lausanne, Switzerland), and antibodies against keratins K17, K75, K28, K82, and K33 (L. Langbein). Donkey anti-guinea pig-Cy3, goat anti-mouse-biotin (Vector Laboratories, Burlingame, CA, USA), and goat anti-rabbit-biotin (Jackson ImmunoResearch, West Grove, PA, USA) were used as secondary antibodies.

BrdU short-term labeling and detection as well as Oil Red O staining were previously described (Schäfer *et al*, 2012).

### Preparation of epidermal whole mounts

Skin was manually peeled off from the mouse tail, cut into approximately 1-cm-long pieces, and incubated in a solution containing 2.5 U/ml dispase (Invitrogen, Carlsbad, CA, USA) in PBS for 2 h at 37°C. Epidermal sheets were separated from the dermis and fixed overnight in 4% PFA or in 95% ethanol/1% acetic acid.

### Morphometrical analysis

Paraffin-embedded back skin was cut longitudinally; tail and eyelid skin were cut transversally to obtain 7-μm sections. Morphometrical analysis of the SG and meibomian gland areas was performed on Adph or hematoxylin/eosin (H&E)-stained serial transverse sections. The gland size of meibomian glands and tail SG was compared at its largest extent. Morphometrical analysis of back skin SG was performed on longitudinal sections. Only areas, where the hair follicles were cut longitudinally, were analyzed. For all analyses, the Openlab 3.1.5 software (Perkin Elmer, Waltham, MA, USA) was used.

### Lipid analysis and quantification

Lipids were extracted from the back skin and analyzed as described previously. The lipids of interest, which contain free fatty acids, triacylglycerol, cholesterol, and cholesterol-and wax esters, were first separated by HPTLC and subsequently quantified by densitometry. In particular, the mentioned lipids were applied in form of thin bands (1 cm in length) automatically on 20 × 10 cm silica HPTLC plates (VWR, Germany). The bands were then separated using a solvent mixture containing n-hexane, diethyl ether, and acetic acid (70:30:1 by volume). After drying, the HPTLC plates were dipped into an aqueous solution containing 10% CuSO_4_ and 8% H_3_PO_4_ (w/v). Heating of the plates to 160°C for 10 min led to visualization of the separated lipids. Visualized lipid bands were quantified using a densitometric scanner (TLC scanner 3; CAMAG, Germany) in the reflectance mode at 595 nm. Quantitative results were obtained by relating the intensities of the separated lipid bands to calibration curves of corresponding standards.

### Chromatin immunoprecipitation

Chromatin lysate was pre-cleared and incubated overnight with antibodies against Nrf2 (Huebner *et al*, 2012), Sprr2 (see above), histone H3 (Abcam, Cambridge, UK), histone H3K4me2, and normal sheep IgG (Upstate/Millipore, Billerica, MA, USA). To analyze protein-bound DNA, primers for qPCR were used (Supplementary Table 2). The percentage of the input that was bound was calculated by the delta Ct method and averaged over at least three experiments.

### Animal experiments

For activation of endogenous Nrf2 in the skin, wild-type mice were topically treated twice a day for 10 consecutive days starting at P0 with 10 mM sulforaphane or 50 mM tBHQ (both from Merck, Darmstadt, Germany) in hydrophilic cream (33%) (Hänseler, Herisau, Switzerland) with DMSO (66%) (vehicle).

EGFR signaling was inhibited by intraperitoneal injection of P18 K5cre-CMVcaNrf2 and control mice with 70 μg Gefitinib (Iressa) (Santa Cruz) per gram body weight once a day for 14 consecutive days. Gefitinib was diluted in DMSO (21%) and olive oil (79%) (vehicle).

Mice were sacrificed by CO_2_ inhalation. Animal maintenance and experiments with animals had been approved by the local veterinary authorities of Zurich, Switzerland.

### Human skin biopsies

Biopsies from affected skin of three patients with TCDD intoxication had been taken for diagnostic purposes after informed consent and with permission from the local ethics committee, Vienna, Austria, and Geneva, Switzerland. Patient 1 initial TCDD level: 144,000 pg/g blood fat, Patient 2 initial TCDD level: 26,000 pg/g blood fat, and Patient 3 initial TCDD level: 108,000 pg/g blood fat. The skin samples had been formalin-fixed and paraffin-embedded and stored in the files of the Department of Dermatology at the Medical University of Vienna since 1998 and at the University of Geneva since 2005. For the current analysis, 5-μm sections were taken and either stained with H&E or further processed for immunohistochemical staining as described above.

### TCDD treatment and siRNA transfection of human foreskin keratinocytes

Primary HFKs were grown in 6-cm dishes in Keratinocyte-SFM (Gibco, Paisley, UK) supplemented with EGF and bovine pituitary extract. They were transfected at 70% confluency with 50 nM 21-mer siRNA targeting NRF2 (Sigma-Aldrich, Buchs, Switzerland) or AHR (Life Technologies, Carlsbad, CA, USA) using INTERFERin (Polyplus, Illkirch, France). After 48 h at 100% confluency, HFKs were washed with PBS and cultured for 48 h in supplement-free Keratinocyte-SFM medium with 10^−8^M TCDD (Sigma-Aldrich) in toluene/DMSO (0.1% final concentration) or toluene/DMSO only.

### *In situ* hybridization

*In situ* hybridization was performed with digoxigenin-labeled sense and antisense probes complementary to bp 91–839 of the murine *Epgn* mRNA (NM_053087.2). For this purpose, the full-length cDNA was amplified by PCR using primers that allowed the introduction of the T7 and SP6 RNA polymerase promoter regions. 400 ng of the purified PCR product was then used to synthesize digoxigenin-labeled RNA probes according to the manufacturer's instructions (DIG RNA Labeling Kit (SP6/T7), Roche Applied Science). *In situ* hybridization was performed as previously described (Brackmann *et al*, 2013). Briefly, 14-μm-thick sections of back skin were post-fixed with 4% PFA and hybridized to the *Epgn* antisense and sense probes at 60°C overnight. The next day, sections were washed and blocked, followed by incubation with an anti-digoxigenin antibody conjugated to alkaline phosphatase (anti-digoxigenin-AP, Fab fragments, Roche Applied Science) overnight. Detection of hybridized probes was performed using NBT/BCIP solution (Roche Applied Science).

### Replicate experiments and statistical analysis

qRT-PCR analyses were performed using cDNA of three or more mice from one litter, three or more pools of different litters, or three or more dishes of cultured cells of one experiment. qRT-PCRs using cDNA from cultured cells were repeated with independent samples. Statistical analysis was performed on samples *N* ≥ 3 using the nonparametric Mann–Whitney *U*-test for non-Gaussian distribution and the Prism 5.0 software (GraphPad Software, La Jolla, CA, USA). Error bars represent standard deviation.
