# Supplementary material for: Activation of Nrf2 in keratinocytes causes chloracne (MADISH)-like skin disease in mice
Source: EMBO Mol Med. 2014 Feb 6;6(4):442–57. doi: 10.1002/emmm.201303281 (PMC3992072; doi:10.1002/emmm.201303281)
Supplement: Supplementary file 1 [file emmm0006-0442-sd1.pdf]

EMBO Molecular Medicine

Activation of Nrf2 in keratinocytes causes chloracne (MADISH)-like skin disease in mice

Matthias Schäfer, Ann-Helen Willrodt, Svitlana Kurinna, Andrea S. Link, Hany Farwanah, Alexandra Geusau, Florian Gruber, Olivier Sorg, Aaron J. Huebner, Dennis R. Roop, Konrad Sandhoff, Jean-Hilaire Saurat, Erwin Tschachler, Marlon R. Schneider, Lutz Langbein, Wilhelm Bloch, Hans-Dietmar Beer, and Sabine Werner

Corresponding author: Matthias Schäfer, ETH Zurich

Review timeline:

Submission date:	05 January 2013
Editorial Decision:	08 January 2013
Appeal Received:	09 January 2013
Editorial Decision:	28 January 2013
Resubmission:	12 July 2013
Editorial Decision:	14 August 2013
Revision received:	10 December 2013
Editorial Decision:	19 December 2013
Revision received:	08 January 2014
Accepted:	08 January 2014

Transaction Report:

(Note: With the exception of the correction of typographical or spelling errors that could be a source of ambiguity, letters and reports are not edited. The original formatting of letters and referee reports may not be reflected in this compilation.)

Editors: Natascha Bushati and Céline Carret

1st Editorial Decision 08 January 2013

Thank you for the submission of your manuscript "Activation of Nrf2 in keratinocytes causes acne-like skin disease in mice". I have now had the opportunity to carefully read your paper and the related literature and I have also discussed it with my colleagues. I am afraid that we concluded that the manuscript is not well suited for publication in EMBO Molecular Medicine and have therefore decided not to proceed with peer review.

Your manuscript reports that prolonged Nrf2 activation in keratinocytes leads to sebaceous gland enlargement, infundibular acanthosis and hyperkeratosis in mice. We appreciate that sebaceous gland hyperplasia is partially attributable to an increase in EGFR signaling by Nrf2 activation.

However, you have previously reported that activation of Nrf2 in keratinocytes causes hyperkeratosis and epidermal thickening, via pathways also described in the present study. In addition, it remains unknown whether Nrf2 and its targets are indeed activated in acne patients.

While your findings will undoubtedly be of interest to investigators in the immediate field, we are not persuaded that your study provides the degree of conceptual advance and pathophysiological relevance we require for publication in EMBO Molecular Medicine.

I am sorry that I could not bring better news this time.

Appeal Received 09 January 2013

We refer to your mail of January 8 with the information that our manuscript will not be sent for outside review. We certainly understand that EMM can only consider a small number of high impact manuscripts, and as an EMBO Member (S.W.) I am particularly careful with the selection of manuscripts that I send to any of the prestigious EMBO journals. We believe, however, that this manuscript has the novelty and quality that would justify a full review process. The data described in this manuscript have raised particular excitement among our colleagues when we presented them at several international meetings. In fact, one of us (Matthias Schäfer) was invited to give a plenary lecture at the upcoming Gordon Research Conference on Epidermal Barrier Function based on these data that he presented at a large meeting in Venice. Furthermore, we have worked in the field of Nrf2 biology since more than ten years and we are well aware of the fact that our findings are entirely novel.

Your decision is based on two major issues – lack of novelty and lack of human data. Please let us comment on these issues.

Lack of novelty: This manuscript provides first evidence for a role of Nrf2 in hair follicle and sebaceous gland biology and pathology. This is completely unexpected, since the major function of Nrf2 is in ROS protection. Second, we identified epigen as the first growth factor target of Nrf2, which not only provides an explanation for the phenotype of our mice, but also suggests a role of this EGF receptor ligand in the pro-tumorigenic effect of activated Nrf2, which is currently heavily investigated. Third, we show that different ratios of Nrf2 target genes cause different pathologies, which is highly relevant with regard to the use of Nrf2 activators in chemoprevention of major human diseases. We agree that Slpi and Sprr2d had previously been identified as Nrf2 targets in the epidermis. However, the effect of their upregulation in the hair follicle is very different from their effect in the epidermis. Therefore, this is also a novel finding of this manuscript. Finally, there are very few mouse lines with acne-like phenotypes and such mice are urgently needed for studies related to this disease. We were therefore approached by several companies and clinicians, who are interested in obtaining these mice.

Lack of human data: This point is well taken. However, acne patients are not biopsied for medical reasons. In contrary, a biopsy would further increase the cosmetic problem of these patients, which is already very severe. Therefore, most acne patients refuse to give biopsies on a voluntary basis. We have discussed this with various dermatologists and they all mentioned that acne patients can usually not be biopsied for ethical reasons. In the rare cases where biopsies are obtained, the disease is rather severe and the lesions are characterized by infection with P. Acnes, which causes severe inflammation. However, we would need biopsies at the early stage of the development of the disease, since our data suggest a role of Nrf2 in the early events of acne development. Lesions at this stage are, however, never biopsied. The problem is aggravated by the fact that we need RNA for the analysis, because immunostaining for nuclear Nrf2 is not reliable, since the available Nrf2 antibodies are not suitable for this purpose. We have nevertheless initiated collaborations with several dermatologists, who will try to obtain such material, but this will take a long time and may even be impossible due to the ethical reasons. Thus, every study on the mechanism of acne development could be rejected for the same reason.

We hope that we have explained the situation and we kindly ask you to re-consider your decision and to send the manuscript for outside review.

2nd Editorial Decision 28 January 2013

Thank you for message regarding our recent decision on your manuscript entitled "Activation of Nrf2 in keratinocytes causes acne-like skin disease in mice". Please accept my sincere apologies for not replying earlier; the reason for this is that the external advisor I initially approached was not available, so I needed to find a replacement.

I do understand your disappointment and thank you for highlighting key aspects of the manuscript. Your points are well-taken and appreciated.

We have now re-discussed your manuscript without prejudice and in addition we have obtained independent external advice from an expert.

The advisor agrees with our original assessment that your paper is lacking the level of conceptual advancement and medical relevance required to make it a high priority for publication in EMBO Molecular Medicine. S/he stated that the study "would fit better in a skin-oriented journal".

In this case, and for these reasons, I am sorry to write that we have decided to uphold our original decision in not sending this manuscript out for in depth peer review.

I also wish to underline that our decision should not be interpreted as being critical of the data quality nor doubting the effort invested in the project, but it is based on its appropriateness for EMBO Molecular Medicine.

Again, I apologize for the delay and am sorry that I could not bring better news.

Resubmission 12 July 2013

In December 2012 we submitted a manuscript to EMM where we described a novel role of the Nrf2 transcription factor in the pathogenesis of acne in mice (entitled "Activation of Nrf2 in keratinocytes causes acne-like skin disease in mice"). Although it is extremely difficult to obtain samples from human acne patients (in particular for ethical reasons), we finally managed to obtain such samples

from collaborators in Austria and Geneva. Together with clinical collaborators we compared the histology of different types of acne in humans with our mouse phenotype and noticed that the latter is most similar to the phenotype seen in patients with chloracne (MADISH). This severe pathology results from exposure to certain halogenated aromatic compounds, in particular dioxin. MADISH became particularly "famous" after the dioxin poisoning of Victor Yushchenko in 2006 and after the Seveso accident in Italy in 1976. We now made the exciting discovery that the same Nrf2 target genes, which we showed to be responsible for the acne phenotype in mice, are also strongly expressed in samples from dioxin-exposed human patients - with a similar spatial expression pattern as in mice. Mechanistically, we show that dioxin-treatment of primary human keratinocytes induces exactly the same genes in vitro. This occurs in an Nrf2-dependent manner as it was abolished upon Nrf2 knock-down. Taken together, we now provide first evidence for a role of Nrf2 and certain novel Nrf2 target genes in that pathogenesis of MADISH.

Since we have now included these exciting human data into our manuscript, and since we also included additional mouse data, we sincerely hope that you will send this strongly revised version for outside review. In this case we would of course upload it through the submission system.

Thank you for considering this new manuscript. We look forward to hearing from you.

3rd Editorial Decision 14 August 2013

Thank you for the submission of your manuscript to EMBO Molecular Medicine. We have now heard back from the three referees whom we asked to evaluate your manuscript. Although the referees find the study to be of potential interest, they also raise a number of concerns that need to be addressed in a major revision of the current manuscript.

From the comments pasted below, you can see that while referee 3 is enthusiastic about the study and only requires to provide statistical information about the data, referee 2 and especially referee 1 have raised a certain number of concerns. These referees request additional experiments to clarify the findings (novelty, significance) and raise the future impact of the study. In our view all requested experiments are valid and would improve the data.

Given these evaluations, I would like to give you the opportunity to revise your manuscript, with the understanding that the referees' concerns must be fully addressed and that acceptance of the manuscript would entail a second round of review. Please note that that it is our journal's policy to allow only a single round of revision, and that acceptance or rejection of the manuscript will therefore depend on the completeness of your response and the satisfaction of the referees with it.

EMBO Molecular Medicine has a "scooping protection" policy, whereby similar findings that are published by others during review or revision are not a criterion for rejection. Should you decide to submit a revised version, I do ask that you get in touch after three months if you have not completed it, to update us on the status.

Please also contact us as soon as possible if similar work is published elsewhere. If other work is published we may not be able to extend the revision period beyond three months.

I look forward to receiving your revised manuscript.

***** Reviewer's comments *****

Referee #1 (Remarks):

This is a potentially interesting paper reporting on the involvement of the Nrf2 transcription factor in chloracne-like skin disease (MADISH). The findings are overall convincing, but there is a novelty

issue that needs to be addressed. The paper would also benefit from further mechanistic/functional insights.

The authors already reported on the consequences of trangenic expression of activated Nrf2 in keratinocytes in this same journal last year. The direct Nrf2 targets that were identified and implicated in acanthosis and hyperkeratosis in the previous paper (SPRR2 and SLP1) are the same that the authors now suggest to be involved in MADISH. However, as acanthosis and hyperkeratosis are also at the basis of this disease, the conceptual novelty of the present findings should be clarified. Along these lines, the induction of the SPRR2 and SLP1 genes by TCDD treatment through a Nrf2-dependent mechanism is interesting but expected, as the connection between TCDD activation of the AHR receptor and Nrf2 expression was also previously reported.

The analysis of MADISH patients-derived material is quite novel and interesting and greater emphasis should be placed on this part of the work. It is very likely that the epidermal alterations of MADISH patients are due to a number of changes in gene expression besides the observed increase in SPRR2 and SLP1 levels. Have the authors examined epigen expression ? More significant functional insights could be provided by global analysis of gene expression in the epidermis of MADISH patients in comparison with keratinocytes plus/minus TCDD treatment and Nrf2 knock-down. This would also solve the novelty issue mentioned above.

Referee #2 (Comments on Novelty/Model System):

The authors demonstrate that constitutive active Nrf2 or chronic exposure to Nrf2 activators has a pathological effect on skin and hair in mouse.
This is of importance for humans because Nrf2 activating compounds are commonly used in skin care products for skin protection and prevention against UVB-induced ROS damages.

Referee #2 (Remarks):

The authors provide a very detailed description of the skin- and hair-related pathologies of the two mouse models that constitutively express either a stronger (i.e., K5Cre-CMVcaNrf2) or a weaker (i.e., K5Cre-caNrf2) Nrf2 activity in keratinocytes.
Using both genetic and pharmacological approaches, Sch‰fer et al. showed that prolonged Nrf2 activation in keratinocytes caused sebaceous gland hyperplasia, infundibular acanthosis, hyperkeratosis, and sebum congestion as a result of the upregulation of a novel Nrf2 target gene called epigen. This eventually led to the cyclic hair loss and hair malformation. Upon ageing of the mice these mice develop cysts, which was caused by an upregulation of Slpi and Sprr2d. These abnormalities were shown to closely resemble human chloracne/MADISH. Overall, this is a very well conducted and thorough work. Most importantly, the authors report a novel and interesting role of Nrf2 activation in the regulation of follicle and sebaceous gland homeostasis as well as its pathological functions in human chloracne/MADISH. Therefore, these findings identify Nrf2 as an attractive pharmacological target for skin protection and cancer prevention, which will warrant advancement in our understanding of MADISH.

However, there is a couple of concerns that need to be addressed.

1) Standard deviation or standard error of the mean was not indicated in all of the real-time qPCR results. The authors indicate (see Legend of Fig. 3) "All qRT-PCRanalyses were performed using cDNA from pools of 2 to 3 mice from one litter and repeated with independent samples from another litter". Nonetheless, an additional sample pool would have allowed the calculation of SD or SEM and would have made statistical analysis meaningful and statistically sound comparisons could been made between the different treatment groups. If samples from a third pool are not available, this would mean repeating a large amount of the work. Otherwise, the authors should at least indicate, in the figure legends, the difference (for instance in %) between the values obtained in the two samples analyzed. Big differences would weaken the conclusions drawn.
2) Page 11 (bottom part) & Figure 7B and 7C. The authors detected strong expression of SLPI and SPRR2 in the epidermis and cyst epithelium of MADISH patients. Immunohistochemical analysis of Nrf2 expression in these tissues seems important for validating (or not) the authors' hypothesis.
3) Page 12 & Figure 8. To better document the molecular mechanism leading to the pathogenesis of

TCDD-induced intoxication, the authors should investigate whether blockade of AHR signalling (either through pharmacological or siRNAs) prevents SLPI- and SPRR2D-mediated hyperkeratosis and hyperplasia in their cultured model.

Minor points:

1) Page 5, 2nd paragraph, Line 6, typographical error: "...infundibulum (INF) (Fig. 1D, lower panel)."
2) Page 5, 2nd paragraph, Line 7, typographical error: "...in the widened INF of K5Cre-CMVcaNrf2 mice (Fig. 1D, upper panel), ..."

Referee #3 (Remarks):

In this manuscript the authors provide new insight into the role of Nrf2 in keratinocytes demonstrating a novel sebaceous (and other) gland phenotype. This is linked at the molecular level to upregulation of multiple growth factors, epigen in particular (a novel Nrf2 target). The paper is very well written providing detailed characterisation of the mouse HF phenotype.

The link to MADISH is intriguing but I wonder if the observed high levels of SPRR2/SLPI as indeed a primary specific link. Are SPRR2/SLPI induced in a general hyperproliferative response? If one looks at a much earlier stage in the human phenotype are the same changes observed (i.e. causative).

In a number of figures qPCR data are presented without statistical validation (Figure 3,4 & 6). The authors state that "cDNA [was used] from pools of 2 to 3 mice from one litter and repeated with independent samples" Essentially these data represent n=2 with no way of confirming statistical significance.

1st Revision - authors' response 10 December 2013

Referee #1 (Remarks):

This is a potentially interesting paper reporting on the involvement of the Nrf2 transcription factor in chloracne-like skin disease (MADISH). The findings are overall convincing, but there is a novelty issue that needs to be addressed. The paper would also benefit from further mechanistic/functional insights.

The authors already reported on the consequences of trangenic expression of activated Nrf2 in keratinocytes in this same journal last year. The direct Nrf2 targets that were identified and implicated in acanthosis and hyperkeratosis in the previous paper (SPRR2 and SLP1) are the same that the authors now suggest to be involved in MADISH. However, as acanthosis and hyperkeratosis are also at the basis of this disease, the conceptual novelty of the present findings should be clarified.

We were pleased to hear that the reviewer finds our results interesting and convincing.

With regard to the novelty we agree with the reviewer that we had previously characterized the epidermal phenotype of the caNrf2 transgenic mice, and we identified Slpi and Sprr2d as novel Nrf2 targets in keratinocytes. In our new study, however, we identified a previously unknown function of Slpi and Sprr2d in the pilosebaceous unit. The upregulation of Slpi and Sprr2d in the infundibulum of K5cre-CMVcaNrf2 mice leads to infundibulum acanthosis and hyperkeratosis and ultimately to cyst formation. We have therefore extended the analysis of this phenotype. Thus, we included pictures of telogen hair follicles of 8w old mice showing infundibulum dilatation (Fig 1F) and we

analyzed incidence and multiplicity of macroscopically visible cysts of 6-month old control and K5cre-CMVcaNrf2 mice (Supporting Information Table S2). Most importantly, we provide evidence that similar changes lead to the development of cysts in the skin of MADISH patients. These data point to a novel function of NRF2 and its target SPRR2D and SLPI in MADISH pathogenesis. This medically highly relevant aspect is completely novel, and we have now extended the analysis of human material and of primary human keratinocytes to further substantiate this finding.

An additional novel finding is the identification of epigen (*Epgn*) as a previously unidentified target of Nrf2 *in vitro* and *in vivo*. Interestingly, this is the first growth factor target of Nrf2, and this finding is therefore also highly relevant with regard to the important role of Nrf2 in cancer. In response to the reviewer`s criticism we performed more experiments on the role of Epgn in the phenotype of our mice and on its potential role in MADISH pathogenesis. We localized *Epgn* mRNA in the infundibulum by *in-situ* hybridization (Fig 3H, Supporting Information Fig S6F) and we found a reduction in keratinocyte proliferation in the infundibulum in gefitinib-injected K5cre-CMVcaNrf2 mice (Fig 5C). Thus, Nrf2-mediated upregulation of Epgn and other EGFR ligands is involved in the development of infundibulum acanthosis and hyperkeratosis in K5cre-CMVcaNrf2 mice. Furthermore, we found strong expression of EPGN in the cysts of MADISH patients (Fig 7E) and upregulation of *EPGN* expression after TCDD stimulation of keratinocytes (Fig 8A). Induction of *EPGN* expression by TCCD involves AHR and NRF2 as revealed by siRNA-mediated knock-down of these proteins (Fig 8D,E). These data unraveled a previously unrecognized function of EPGN in MADISH pathogenesis.

Along these lines, the induction of the SPRR2 and SLP1 genes by TCDD treatment through a Nrf2-dependent mechanism is interesting but expected, as the connection between TCDD activation of the AHR receptor and Nrf2 expression was also previously reported.

It had indeed been shown in several cell lines that TCDD stimulation activates NRF2, leading to upregulation of NRF2 and classical NRF2 target genes. However, to the best of our knowledge, this has so far not been reported for human keratinocytes and these are the cells in MADISH patients, which are responsible for cyst formation. It is important to show this in keratinocytes, since targets of transcription factors (including Ahr and Nrf2) are tissue and cell-type specific. Most importantly, we show for the first time that TCDD stimulation leads to upregulation of SLPI, SPRR2D and EPGN, and our siRNA experiments demonstrate that this is mediated by NRF2 activation. Most importantly, we provide first evidence for an important role of the TCCD-AHR-NRF2 axis in the skin *in vivo*.

Following the suggestion of reviewer 2 we performed siRNA-mediated knock-down of AHR in primary human keratinocytes. The results of the experiment revealed that TCDD activates the novel NRF2 targets *SLPI*, *SPRR2D* and *EPGN* in keratinocytes via AHR activation (Fig 8E and Supporting Information Fig S7B), and that both AHR and NRF2 are required for the TCDD-mediated activation of these genes. These are all novel data, which provide insight into the molecular events that occur in keratinocytes after TCDD stimulation and into the mechanisms underlying MADISH pathogenesis.

The analysis of MADISH patients-derived material is quite novel and interesting and greater emphasis should be placed on this part of the work. It is very likely that the epidermal alterations of MADISH patients are due to a number of changes in gene expression besides the observed increase in SPRR2 and SLP1 levels. Have the authors examined epigen expression ?

As requested by the reviewer, we extended the analysis of human samples (which are extremely difficult to obtain!) and we place greater emphasis on the human part of the work:

1. We analyzed expression of *EPGN* in TCDD stimulated keratinocytes and we observed *EPGN* upregulation (Fig 8A). Furthermore, siRNA-mediated knock-down of NRF2 or AHR in primary human keratinocytes strongly reduced TCDD-induced upregulation of *EPGN* (Fig 8D, E), demonstrating that TCDD upregulates *EPGN* via AHR and NRF2 activation.

2. We performed EPGN immunohistochemistry staining on skin samples of MADISH patients, which showed a strong EPGN expression in the cyst epithelium (Fig 7E). We also detected a strong expression of the "classical" NRF2 target NQO1 in the cyst epithelium, indicating NRF2 activation (Fig 7D).

In addition, we extended the analysis on the role of epigen in the murine phenotype: 1.) We analyzed the expression of *Epgn* in wild-type mice by *in-situ* hybridization, which revealed expression in the epidermis, hair follicles and sebaceous glands (Fig 3H). Thus, epigen is indeed expressed in the cells that are affected by TCDD, resulting in MADISH in humans.

2.) To find out whether Epgn upregulation in K5cre-CMVcaNrf2 mice is also responsible for INF acanthosis, we analyzed proliferation in vehicle- and gefitinib- injected control and K5cre-CMVcaNrf2 mice. Immunohistochemistry for PCNA revealed a significant reduction in the number of proliferating cells in the infundibulum of gefitinib-injected compared to vehicle-injected K5cre-CMVcaNrf2 mice (Fig 5C).

These results demonstrate that Epgn upregulation not only increases sebaceous gland hyperplasia, but together with other EGFR ligands it also stimulates proliferation of keratinocytes of the infundibulum, which ultimately results in infundibulum hyperplasia. These data further suggest that TCDD-mediated upregulation of EPGN in keratinocytes via NRF2 activation contributes to hair follicle hyperplasia and cyst formation in MADISH patients.

More significant functional insights could be provided by global analysis of gene expression in the epidermis of MADISH patients in comparison with keratinocytes plus/minus TCDD treatment and Nrf2 knock-down. This would also solve the novelty issue mentioned above.

We agree that this would be an interesting and important experiment. However, this needs to be performed at an early stage of the disease to unravel the initial molecular alterations that ultimately lead to the skin phenotype. If performed at a late stage, the observed alterations are secondary to changes that had occurred long before when the toxic insult was still present. Unfortunately, biopsies from MADISH patients are extremely rare, and only available at later stages of the disease. We collaborate with several hospitals that had previously treated such patients (see our list of authors), but samples from newly exposed patients are not available, since they only present to the hospital when the symptoms appear (and thus after the exposure). In fact, we could not even obtain frozen samples from late stage patients that could be used for microarray analysis – it is only possible to obtain a few sections from these very rare samples. It is furthermore not possible to perform global analysis in TCDD treated mice, since this is not leading to an acne phenotype, most likely because TCDD is differently metabolized in rodents compared to men.

However, we had previously performed microarray analysis using RNA from skin of P2.5 K5cre-CMVcaNrf2 and control mice. The only genes, which were significantly upregulated and which could explain the acanthosis and hyperkeratosis phenotype of our mice, are *Slpi*, *Sprr2d* and *Epgn*. We added data obtained with RNA from preputial glands of K5cre-CMVcaNrf2 mice, which also became hyperkeratotic upon ageing, and we found a upregulation of all three genes (Supporting information Fig S6G). This finding further documents that upregulation of these genes is generally associated with a hyperkeratotic phenotype.

It has previously been described that TCDD induces expression of a variety of genes of the epidermal differentiation complex and their upregulation was suggested to be involved in cyst formation of MADISH patients (Sutter et al. 2011, Toxicol. Sciences). We had previously analyzed expression of *Sprr1a* and we have now analyzed *Sprr2a*, *Lce3a*, and *S100A6*. These genes were highly upregulated in human keratinocytes after TCDD stimulation (published by Sutter et al. 2011, Toxicol. Sciences). However, none of these genes showed an increase in P32 K5cre-CMVcaNrf2 mice (Supporting Information Fig S6H) (Schäfer et al., 2012). This further supports our hypothesis that Slpi, Sprr2d and Epgn are the major drivers of the phenotype in K5cre-CMVcaNrf2 mice and this is obviously sufficient for cyst formation.

Altogether, our data provide strong evidence for an important and previously unrecognized role of NRF2 in the development of MADISH and they demonstrate that the direct NRF2 targets SPRR2D, SLPI and EPGN are responsible for cyst formation. While SPRR2D and other EDC targets have

© EMBO

been implicated in MADISH pathogenesis before, SLPI and EPGN are two novel TCCD target genes, which are first described in this manuscript. Thus, we unraveled a novel TCCD-AHR-NRF2-SPRR2/SLPI/EPGN axis in the pathogenesis of MADISH.

Referee #2 (Comments on Novelty/Model System):

The authors demonstrate that constitutive active Nrf2 or chronic exposure to Nrf2 activators has a pathological effect on skin and hair in mouse.

This is of importance for humans because Nrf2 activating compounds are commonly used in skin care products for skin protection and prevention against UVB-induced ROS damages.

Referee #2 (Remarks):

The authors provide a very detailed description of the skin- and hair-related pathologies of the two mouse models that constitutively express either a stronger (i.e., K5Cre-CMVcaNrf2) or a weaker (i.e., K5Cre-caNrf2) Nrf2 activity in keratinocytes.

Using both genetic and pharmacological approaches, Schäfer et al. showed that prolonged Nrf2 activation in keratinocytes caused sebaceous gland hyperplasia, infundibular acanthosis, hyperkeratosis, and sebum congestion as a result of the upregulation of a novel Nrf2 target gene called epigen. This eventually led to the cyclic hair loss and hair malformation. Upon ageing of the mice these mice develop cysts, which was caused by an upregulation of Slpi and Sprr2d. These abnormalities were shown to closely resemble human chloracne/MADISH. Overall, this is a very well conducted and thorough work. Most importantly, the authors report a novel and interesting role of Nrf2 activation in the regulation of follicle and sebaceous gland homeostasis as well as its pathological functions in human chloracne/MADISH. Therefore, these findings identify Nrf2 as an attractive pharmacological target for skin protection and cancer prevention, which will warrant advancement in our understanding of MADISH.

However, there is a couple of concerns that need to be addressed.

1) Standard deviation or standard error of the mean was not indicated in all of the real-time qPCR results. The authors indicate (see Legend of Fig. 3) "All qRT-PCRanalyses were performed using cDNA from pools of 2 to 3 mice from one litter and repeated with independent samples from another litter". Nonetheless, an additional sample pool would have allowed the calculation of SD or SEM and would have made statistical analysis meaningful and statistically sound comparisons could been made between the different treatment groups. If samples from a third pool are not available, this would mean repeating a large amount of the work. Otherwise, the authors should at least indicate, in the figure legends, the difference (for instance in %) between the values obtained in the two samples analyzed. Big differences would weaken the conclusions drawn.

This point is well taken. We repeated all qPCR analyses shown in in the main figures with RNAs from three or more pools of mice from different litters or from three or more individual mice. The SD is now shown in the graphs and statistical analyses were performed.

In the Supporting Information figures we integrated qPCR data obtained with RNAs from a second independent litter or we also we repeated qPCRs with RNAs from three or more individual mice.

2) Page 11 (bottom part) & Figure 7B and 7C. The authors detected strong expression of SLPI and SPRR2 in the epidermis and cyst epithelium of MADISH patients. Immunohistochemical analysis of Nrf2 expression in these tissues seems important for validating (or not) the authors' hypothesis.

Unfortunately, it is not possible to perform immunohistochemistry analysis of NRF2 on human sections, since there is no good antibody available, which would allow the detection of human NRF2

in either the cytoplasm or in the nucleus (where it is located upon activation). We therefore performed immunohistochemistry analysis of the classical NRF2 target NQO1 using sections of MADISH patients (Fig 7D). We observed a strong staining in differentiated keratinocytes of the epidermis and cyst epithelium, further suggesting NRF2 activation in MADISH patients.

3) Page 12 & Figure 8. To better document the molecular mechanism leading to the pathogenesis of TCDD-induced intoxication, the authors should investigate whether blockade of AHR signalling (either through pharmacological or siRNAs) prevents SLPI- and SPRR2D-mediated hyperkeratosis and hyperplasia in their cultured model.

This is indeed an interesting question. We therefore transfected human foreskin keratinocytes with two different siRNAs against AHR. Both lead to a significant reduction of *AHR* and *CYP1A1* expression (Supporting Information Fig 7B). Most importantly, both siRNAs inhibited TCDD-mediated upregulation of *NQO1*, *SLPI*, *SPRR2D* and *EPGN*, demonstrating that TCDD activates NRF2 and its target genes by AHR activation.

Unfortunately, however, it is not possible to study the consequences of AHR knock-down on acanthosis and hyperkeratosis in our culture model, since keratinocytes do not show appropriate differentiation and stratification in 2D cultures. However, since we demonstrated an important role of SLPI and SPRR2D in the development of hyperkeratosis and hyperplasia, we believe that the analysis of their expression upon AHR downregulation sufficiently addresses this point.

Minor points:

1) Page 5, 2nd paragraph, Line 6, typographical error: "...infundibulum (INF) (Fig. 1D, lower panel)."

2) Page 5, 2nd paragraph, Line 7, typographical error: "...in the widened INF of K5Cre-CMVcaNrf2 mice (Fig. 1D, upper panel), ..."

We corrected both typos.

Referee #3 (Remarks):

In this manuscript the authors provide new insight into the role of Nrf2 in keratinocytes demonstrating a novel sebaceous (and other) gland phenotype. This is linked at the molecular level to upregulation of multiple growth factors, epigen in particular (a novel Nrf2 target). The paper is very well written providing detailed characterisation of the mouse HF phenotype.

The link to MADISH is intriguing but I wonder if the observed high levels of SPRR2/SLPI as indeed a primary specific link. Are SPRR2/SLPI induced in a general hyperproliferative response?

This is an important point. We therefore analyzed the expression of SPRR2 and SLPI on sections of human basal cell carcinoma. Both proteins were strongly expressed in differentiated keratinocytes in normal skin, but the staining was weaker in several sections of basal cell carcinomas, which represent a hyperproliferative condition (Supporting Information Fig S7C). This result demonstrates that upregulation of SPRR2/SLPI is not a general hyperproliferative response.

To further address the issue of specificity, we analyzed microarray data that had been obtained with

RNA from skin of P2.5 K5cre-CMVcaNrf2 and control mice. The only genes, which were significantly upregulated and which could explain the acanthosis and hyperkeratosis phenotype of our mice are *Slpi*, *Sprr2d* and *Epgn*. It was previously described that TCDD induces expression of a variety of genes of the epidermal differentiation complex and their upregulation could be involved in cyst formation of MADISH patients (Sutter et al. 2011, Toxicol. Sciences). We had previously analyzed expression of *Sprr1a* and we have now analyzed *Sprr2a*, *Lce3a*, and *S100A6*. These genes were highly upregulated in human keratinocytes after TCDD stimulation (published by Sutter et al. 2011, Toxicol. Sciences). However, none of these genes showed an increase in P32 K5cre-CMVcaNrf2 mice (Supporting Information Fig S6H) (Schäfer et al., 2012). This further supports our hypothesis that Slpi, Sprr2d and Epgn are the major drivers of the phenotype in K5cre-CMVcaNrf2 mice and this is obviously sufficient for cyst formation.

If one looks at a much earlier stage in the human phenotype are the same changes observed (i.e. causative).

This is indeed a very interesting question, which we would love to address. Unfortunately, biopsies from MADISH patients are extremely rare and only available at later stages of the disease. We collaborate with several hospitals that had previously treated such patients (see our list of authors), but samples from newly exposed patients are not available, since they only present to the hospital when the symptoms appear (and thus after the exposure).

In a number of figures qPCR data are presented without statistical validation (Figure 3,4 & 6). The authors state that "cDNA [was used] from pools of 2 to 3 mice from one litter and repeated with independent samples" Essentially these data represent n=2 with no way of confirming statistical significance.

This point is well taken. We repeated all qPCR analyses shown in the main figures with RNAs from three or more pools of mice from different litters or from three or more individual mice. The SD is now shown in the graphs and statistical analyses were performed.

In the Supplemental figures we integrated qPCR data of a second independent litter.

4th Editorial Decision 19 December 2013

Thank you for the submission of your revised manuscript to EMBO Molecular Medicine. We have now received the enclosed reports from the referees that were asked to re-assess it. As you will see the reviewers are now supportive and I am very pleased to inform you that we will be able to accept your manuscript pending final editorial amendments.

I look forward to reading a new revised version of your manuscript as soon as possible.

***** Reviewer's comments *****

Referee #1 (Remarks):

The authors have addressed all my concerns, novelty issue included. I am therefore happy to recommend publication of the paper in its present form.

Referee #2 (Remarks):

Much additional work has been done to meet the reviewers' queries.
The results obtained and incorporated in the manuscript have significantly improved the quality of this nice piece of work.
