# Supplementary material for: Activation of Nrf2 in keratinocytes causes chloracne (MADISH)-like skin disease in mice
Source: EMBO Mol Med. 2014 Feb 6;6(4):442–57. doi: 10.1002/emmm.201303281 (PMC3992072; doi:10.1002/emmm.201303281)
Supplement: Supplementary file 2 [file emmm0006-0442-sd2.pdf]

FIGURES AND TABLES

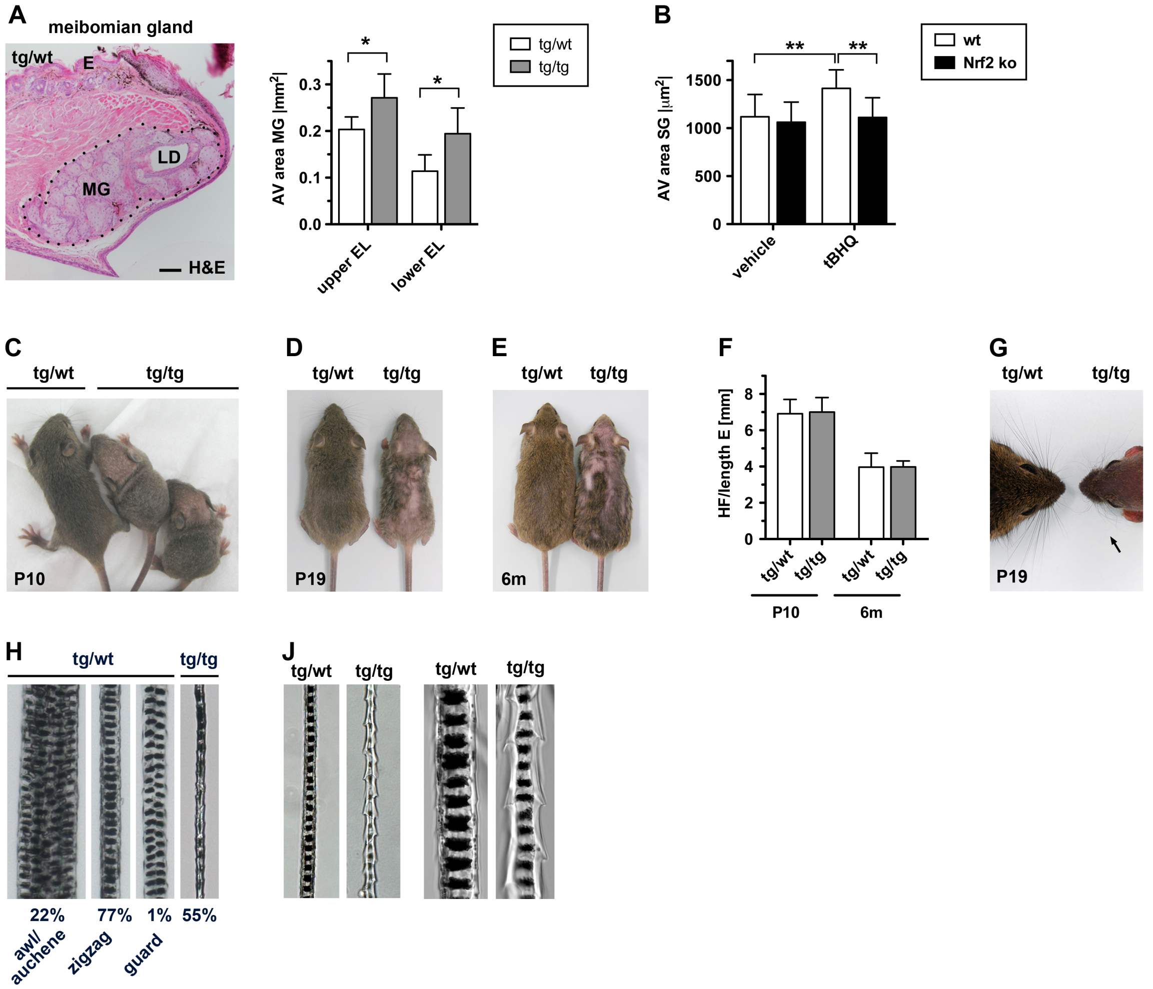

Supporting Information Fig S1: Pilosebaceous phenotype of K5cre-CMVcaNrf2 mice

(**A**) Left: Transverse section of a tg/wt eyelid with meibomian gland (indicated by dotted line). Right: Meibomian gland area in lower (N=6/5, *P=0.0317) and upper eyelid (N=6/5; *P=0.0317) of tg/wt and tg/tg mice. Values are shown as the mean with SD. P-values were calculated by Mann-Whitney-U test. Scale bar: 100µm. (**B**) Morphometric analysis of SG area of wt and *Nrf2* ko mice topically treated with vehicle or tBHQ. Note the lack of tBHQ-induced increase in SG area in *Nrf2* ko mice. The relatively mild SG enlargement in tBHQ-treated wt mice compared to the mice shown in Fig. 1C is due to the backcrossing of these mice into the C57BL/6 background. Wt tBHQ vs. wt vehicle (N=8/17, **P=0.0057), *Nrf2* ko tBHQ vs. wt tBHQ (N=17/8, **P=0.0039). Values are

shown as the mean with SD. P-values were calculated by Mann-Whitney-U test. (**C-E**) Macroscopic appearance of tg/tg and tg/wt mice at P10 (C), P19 (D) and 6m (E). Note bald areas on head, trunk and tail in tg/tg mice. (**F**) Hair follicle number per length epidermis in P10 (N=5/6) and 6m old (N=7) tg/wt and tg/tg mice. Values are shown as the mean with SD. (**G**) Snout of P19 tg/wt and tg/tg mice. Note thin and curly whiskers in tg/tg mice (arrow). (**H**) Pelage hairs of tg/wt and tg/tg mice. Tg/wt mice have 22% awl, 77% zigzag and 1% guard hairs. 55% of the tg/tg pelage hairs are thin and slightly curly (N=5, n>300). (**J**) Pelage hairs of tg/wt and tg/tg mice in overview (left panel) and close up (right panel), showing perpendicular cuticle cells in tg/tg mice. AV, average; E, epidermis; LD, luminal duct; MG; meibomian gland.
