# Supplementary material for: Activation of Nrf2 in keratinocytes causes chloracne (MADISH)-like skin disease in mice
Source: EMBO Mol Med. 2014 Feb 6;6(4):442–57. doi: 10.1002/emmm.201303281 (PMC3992072; doi:10.1002/emmm.201303281)
Supplement: Supplementary file 3 [file emmm0006-0442-sd3.pdf]

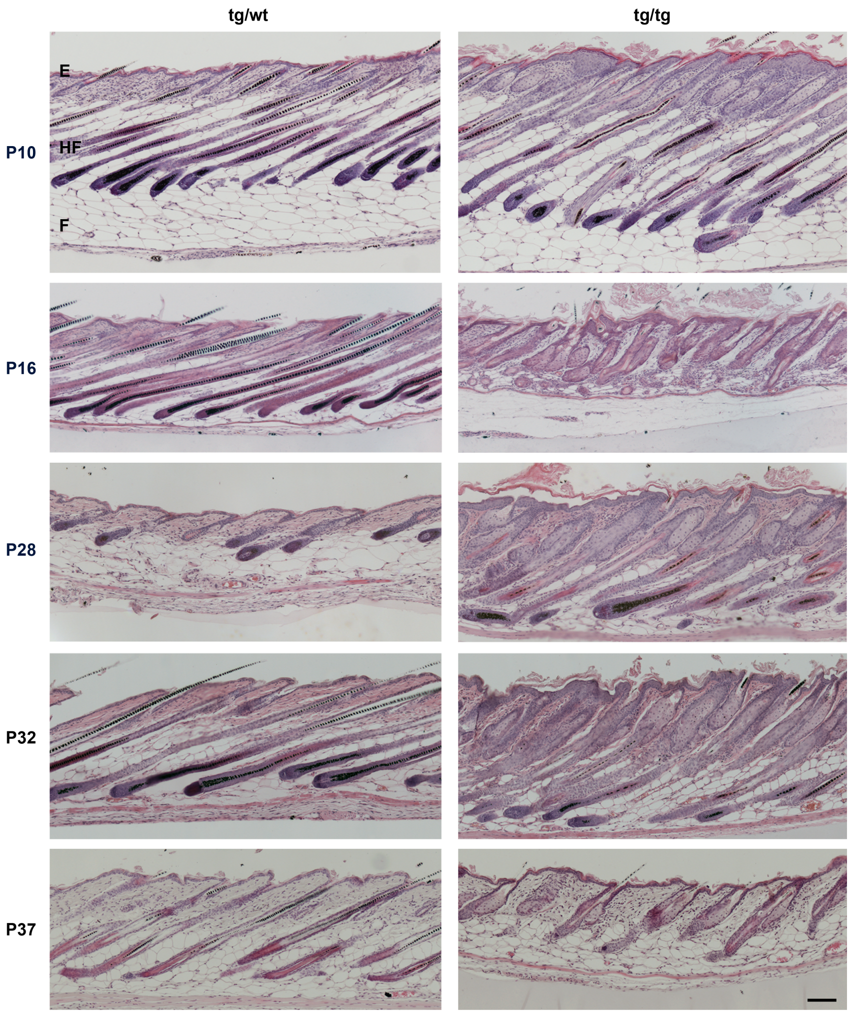

Supporting Information Fig S2: K5cre-CMVcaNrf2 mice have a shift in the pelage hair cycle

Longitudinal sections of back skin from control (tg/wt) and K5cre-CMVcaNrf2 (tg/tg) mice at P10, P16, P28, P32, and P37. At P10 hair follicles of tg/wt and tg/tg mice are both in anagen, but tg/tg hair follicles are longer. At P16 tg/wt hair follicles are still in anagen, tg/tg hair follicles are in late catagen. At P28 tg/wt hair follicles enter the anagen phase, tg/tg hair follicles are in mid-anagen. At P32 hair follicles of both tg/wt and tg/tg mice are in mid/late-anagen and show approximately the same length. At P37 tg/wt hair follicles enter the catagen phase, tg/tg hair follicles are already in mid-catagen. Scale bar: 100μm. E, epidermis; F, subcutaneous fat; HF, hair follicle.
