# Supplementary material for: Activation of Nrf2 in keratinocytes causes chloracne (MADISH)-like skin disease in mice
Source: EMBO Mol Med. 2014 Feb 6;6(4):442–57. doi: 10.1002/emmm.201303281 (PMC3992072; doi:10.1002/emmm.201303281)
Supplement: Supplementary file 4 [file emmm0006-0442-sd4.pdf]

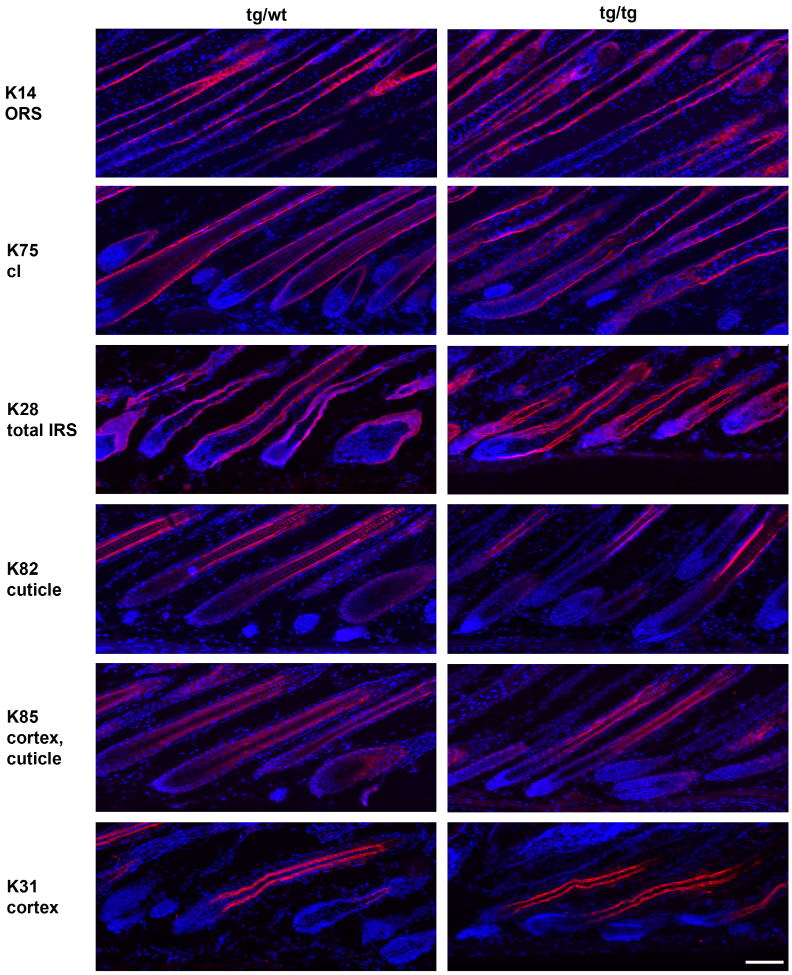

Supporting Information Fig S3: Normal expression of differentiation markers in the lower part of the hair follicles of K5cre-CMVcaNrf2 mice

Immunofluorescence staining of longitudinal sections from back skin of control (tg/wt) and K5cre-CMVcaNrf2 (tg/tg) mice for K14 (outer root sheath, ORS), K75 (companion layer, cl), K28 (total inner root sheath, IRS), K82 (cuticle), K85 (cortex and cuticle) and K31 (cortex). No difference in expression of the analysed marker proteins was observed between hair follicles of tg/wt and tg/tg mice. Scale bar: 50µm.
