# Supplementary material for: Activation of Nrf2 in keratinocytes causes chloracne (MADISH)-like skin disease in mice
Source: EMBO Mol Med. 2014 Feb 6;6(4):442–57. doi: 10.1002/emmm.201303281 (PMC3992072; doi:10.1002/emmm.201303281)
Supplement: Supplementary file 5 [file emmm0006-0442-sd5.pdf]

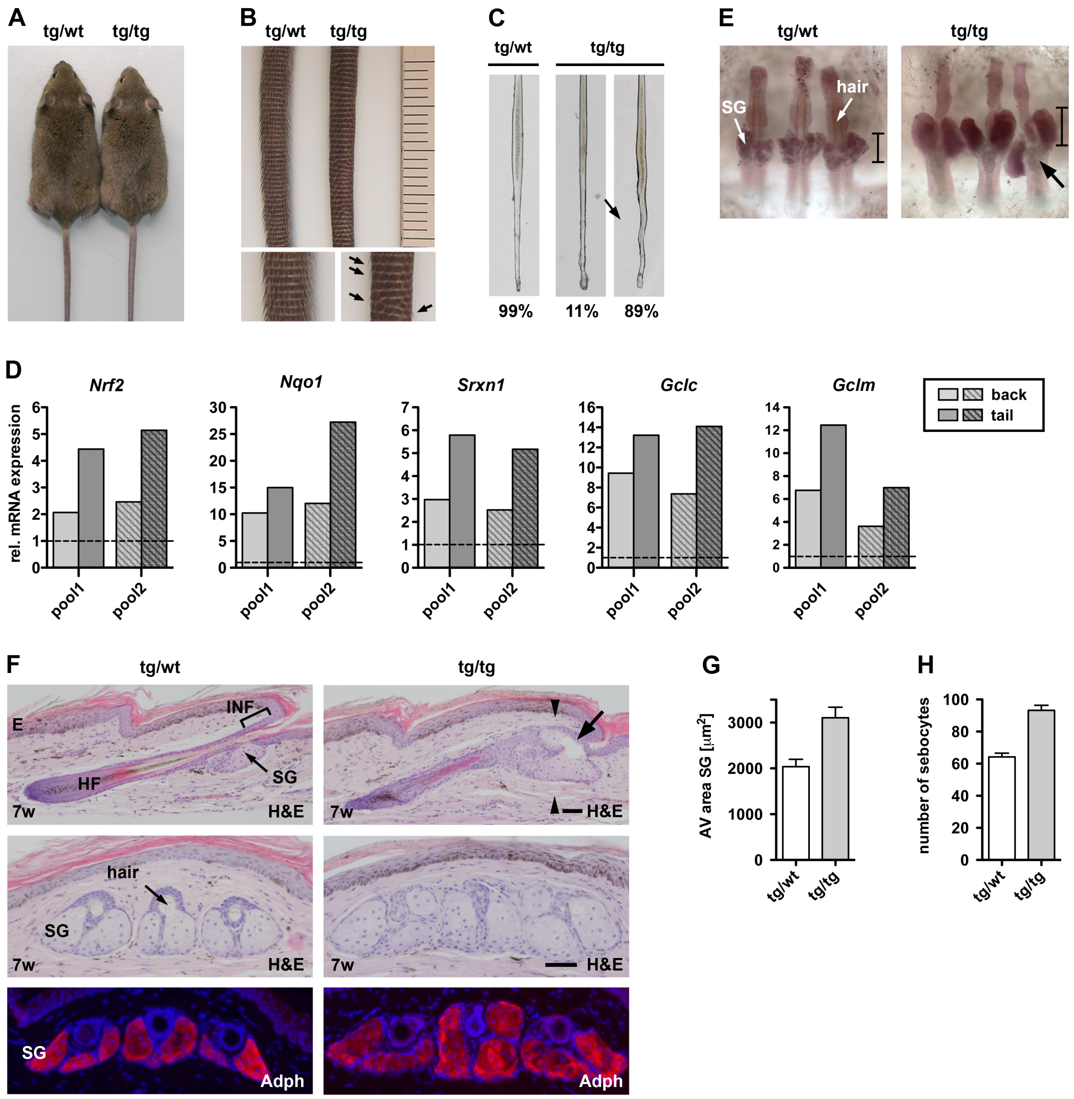

Supporting Information Fig S4: Pilosebaceous phenotype of K5cre-caNrf2 mice

(**A,B**) Control (tg/wt) and K5cre-caNrf2 (tg/tg) mice at 7 weeks of age in overview (A) and close-up of the tail (B). Note the reduction of tail hairs in tg/tg mice. Arrows in (B) point to remaining hairs in the tail. (**C**) Tail hairs of tg/wt and tg/tg mice. In tg/tg mice, 89% of tail hairs are curly in the lower part (indicated by arrow), while 11% have a normal morphology (N=6, n>300). (**D**) qRT-PCR of *Nrf2* (endogenous *Nrf2* and transgene-derived *caNrf2*), *Nqo1*, *Srxn1, Gclc*, and *Gclm* relative to *Gapdh* using back skin (light grey) and tail skin RNA (dark grey) of tg/wt and tg/tg mice. Expression in tg/wt mice was arbitrarily set as 1, indicated by dashed line. Results obtained with RNAs pooled from two to three mice from two independent litters (pool1 and pool2) are shown.

Note the stronger overexpression of all genes in tail skin compared to back skin in tg/tg compared to tg/wt mice. (**E**) Whole mount of tail epidermis and pilosebaceous unit stained with Haemalaun. Note enlarged sebaceous glands (SG, indicated by vertical bar) and sebum retention in the infundibulum (INF) (indicated by arrow) of tg/tg mice. (**F**) H&E staining of longitudinal (upper panel) and transverse tail skin sections (middle panel) and immunofluorescence for Adph of transverse tail skin sections (lower panel) of 7w old mice. Tg/tg mice have enlarged SG and INF dilatation (indicated by arrow in upper panel). Arrowheads in upper panel indicate approximate position of transverse section shown in middle panel. Scale bars: 50µm. (**G**,**H**) SG area (G, N=3) and number of sebocytes (H, N=3) of tg/wt and tg/tg tail skin. Values are shown as the mean with SD. AV, average; E, epidermis; HF, hair follicle; INF, infundibulum; SG, sebaceous gland.
