# Supplementary material for: Activation of Nrf2 in keratinocytes causes chloracne (MADISH)-like skin disease in mice
Source: EMBO Mol Med. 2014 Feb 6;6(4):442–57. doi: 10.1002/emmm.201303281 (PMC3992072; doi:10.1002/emmm.201303281)
Supplement: Supplementary file 6 [file emmm0006-0442-sd6.pdf]

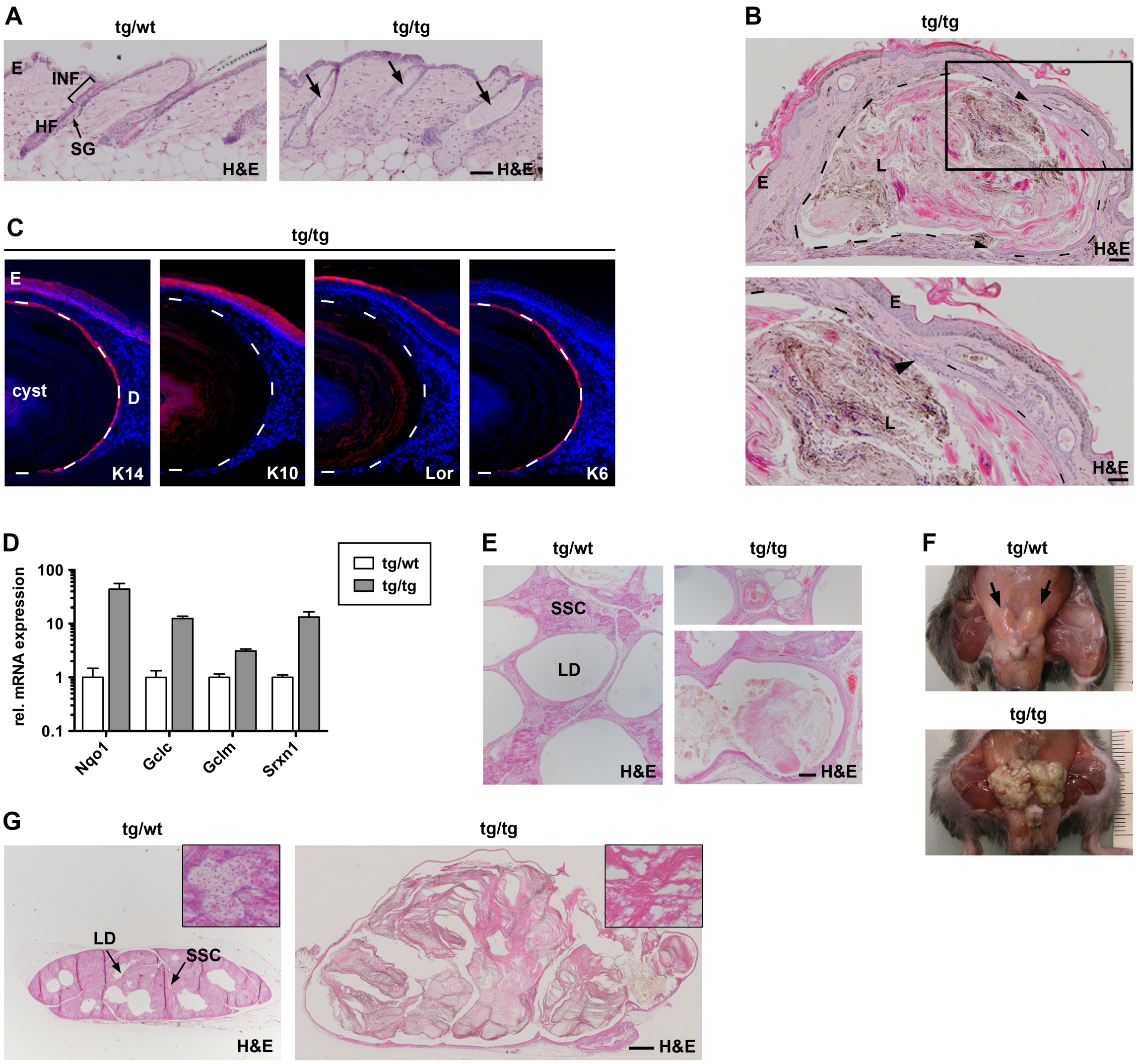

Supporting Information Fig S5: Histological and molecular analysis of skin and preputial glands of aged K5cre-CMVcaNrf2 mice

(**A**) H&E staining of longitudinal back skin sections of 1 year (1y) old mice. Back skin hair follicles of tg/tg mice show severe INF dilatation and keratinization (indicated by arrows). Scale bar: 50µm. (**B**) H&E staining of a large tail skin cyst in overview (upper picture) and close-up (lower picture). The area shown in the close-up is indicated by a rectangle in the upper picture. Note ruptured epithelium indicated by arrowhead. Dashed line indicates cyst boundary. Scale bars: 100µm (upper picture), 50µm (lower picture). (**C**) Immunofluorescence for K14, K10, loricrin and K6 (red), counterstained with Hoechst (blue). Note expression of K14, K10 and loricrin in the interfollicular epidermis

and of K14 and K6 in the cyst epithelium (indicated by dashed line). Scale bar: 30µm. (**D**) qRT-PCR of *Nqo1*, *Gclc*, *Gclm* and *Srxn1* relative to *Gapdh* using RNA from isolated preputial glands of 1 year-old tg/tg and tg/wt mice. Expression in tg/wt mice was arbitrarily set as 1. All genes show elevated expression levels in preputial glands of tg/tg mice compared to tg/wt mice (N=3). Values are shown as the mean with SD. (**E**) H&E staining of preputial gland transverse sections of tg/wt and tg/tg mice. Preputial glands of tg/tg mice have a hyperkeratotic luminal duct. Scale bar: 100µm. (**F**) Pictures of the opened abdomen (upper panel) showing highly enlarged preputial glands in tg/tg mice. (**G**) H&E staining of preputial gland transverse sections of tg/wt and tg/tg K5cre-CMVcaNrf2 mice (F2 C57BL/6 back-crossing). Tg/tg mice have enlarged, keratinized preputial glands, which are also filled with sebum. Inset picture shows a representative close-up of the preputial gland. Scale bar: 500µm. SSC, sebum secretory cells; LD, luminal duct.
