# Supplementary material for: Activation of Nrf2 in keratinocytes causes chloracne (MADISH)-like skin disease in mice
Source: EMBO Mol Med. 2014 Feb 6;6(4):442–57. doi: 10.1002/emmm.201303281 (PMC3992072; doi:10.1002/emmm.201303281)
Supplement: Supplementary file 7 [file emmm0006-0442-sd7.pdf]

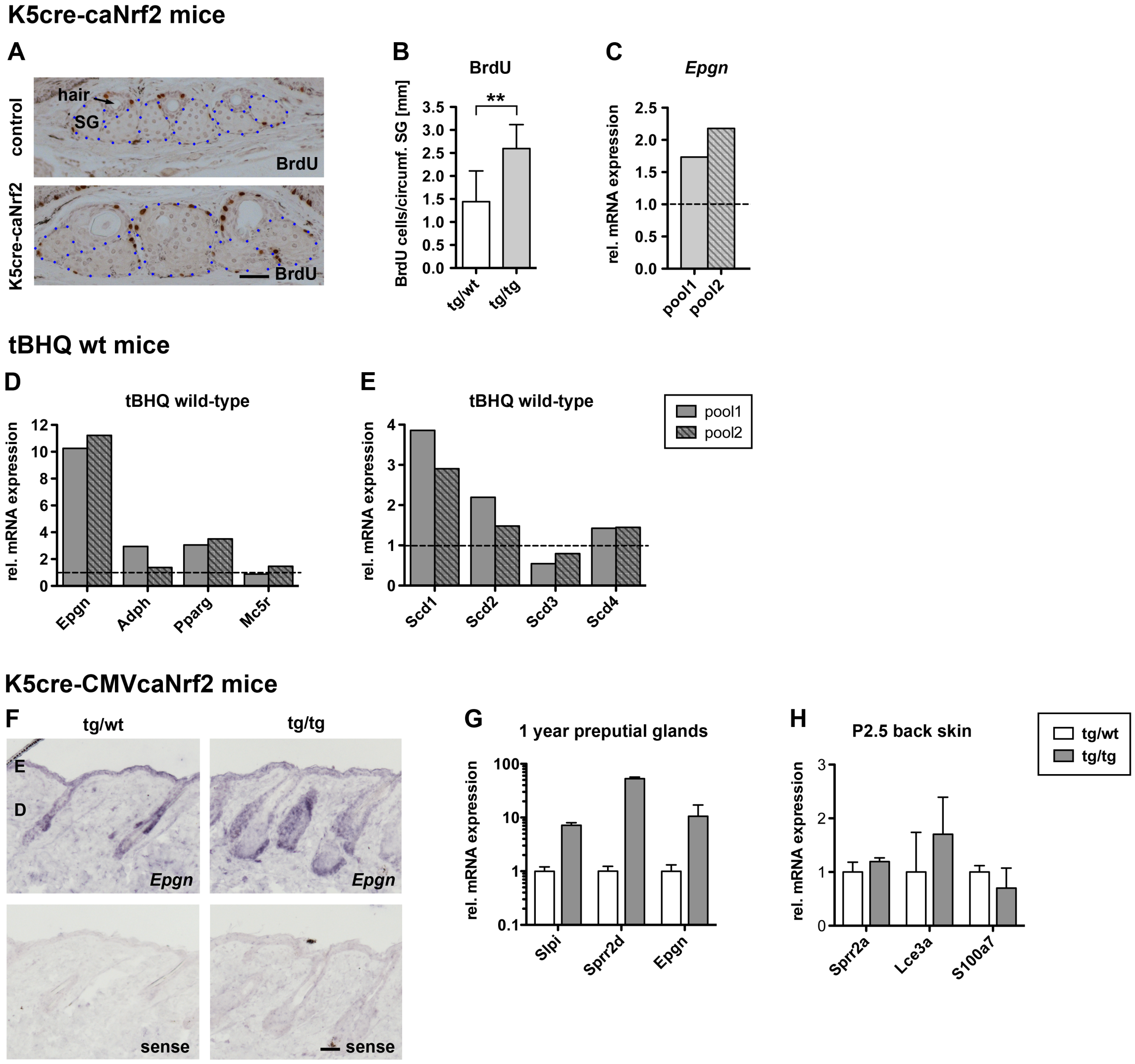

Supporting Information Fig S6: Nrf2 activation increases sebocyte proliferation by induction of epigen expression and affects sebocyte differentiation

(**A**) BrdU staining of transverse tail skin sections of control and K5cre-caNrf2 mice. Note the increase in BrdU positive peripheral sebocyte progenitors (indicated by blue dots) and ORS of the hair follicles in K5cre-caNrf2 mice. Scale bar: 100µm. (**B**) Quantification of BrdU positive sebocyte progenitor cells per circumference of the sebaceous gland in control (tg/wt) and K5cre-caNrf2 (tg/tg) mice (N=5/6, **P=0.0043). Measurements were performed on serial sections, and the SG circumferences at the largest extent were used for the statistical analysis. Values are shown as the mean with SD. P-values were calculated by Mann-Whitney-U test. (**C**) qRT-PCR of *Epgn* relative to *Gapdh* using

RNAs from skin of control (tg/wt) and K5cre-caNrf2 (tg/tg) mice. Expression of tg/wt mice was arbitrarily set as 1. Results obtained with RNAs pooled from two to three mice from two independent litters (pool1 and pool2) are shown. (**D,E**) qRT-PCR of *Epgn*, *Adph*, *Pparg*, *Mc5r* (D) and *Scd1*, *2*, *3*, *4* (E) relative to *Gapdh* using RNAs from back skin of tBHQ-treated and vehicle-treated wt mice. Results obtained with RNAs pooled from three mice from two independent litters (pool1 and pool2) are shown. Expression in vehicle-treated mice was arbitrarily set as 1, indicated by dashed line. (**F**) *In-situ* hybridization on 8w old tg/wt and tg/tg back skin sections using *Epgn* sense (upper panel) and antisense probes (lower panel). Note strong staining in interfollicular epidermis and pilosebaceous unit on tg/wt and tg/tg section hybridized with the *Epgn* antisense probe. Scale bar: 50μm. (**G**) qRT-PCR of *Slpi*, *Sprr2d*, and *Epgn* relative to *Gapdh* using RNA from isolated preputial glands of 1 year-old tg/tg and tg/wt mice. Expression in tg/wt mice was arbitrarily set as 1. All genes show elevated expression levels in preputial glands of tg/tg mice compared to tg/wt mice (N=4/3). Values are shown as the mean with SD. (**H**) qRT-PCR of *Sprr2a*, *Lce3a*, and *S100a6* relative to *Gapdh* using RNA from isolated preputial glands of P2.5 tg/tg and tg/wt mice. Expression in tg/wt mice was arbitrarily set as 1 (N=3). Values are shown as the mean with SD.
