# Supplementary material for: Activation of Nrf2 in keratinocytes causes chloracne (MADISH)-like skin disease in mice
Source: EMBO Mol Med. 2014 Feb 6;6(4):442–57. doi: 10.1002/emmm.201303281 (PMC3992072; doi:10.1002/emmm.201303281)
Supplement: Supplementary file 8 [file emmm0006-0442-sd8.pdf]

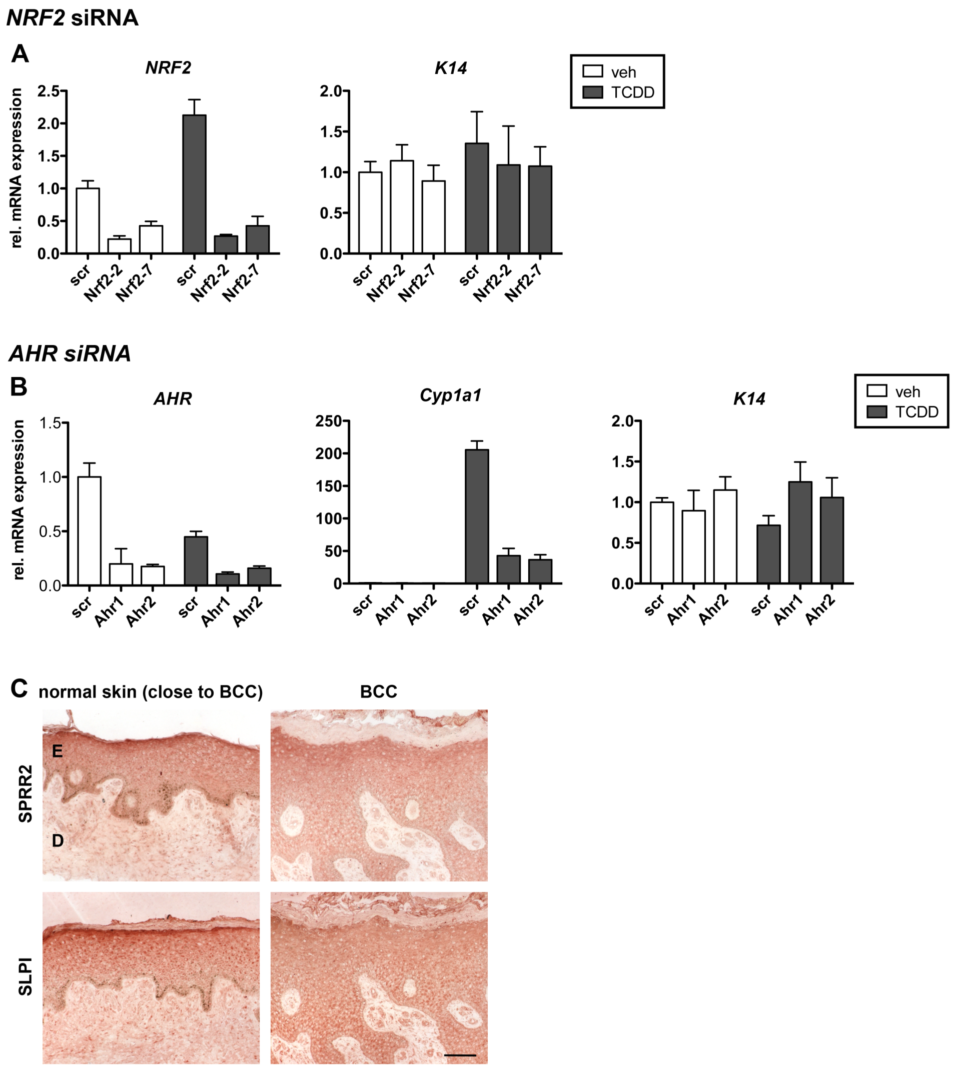

Supporting Information Fig S7: siRNA-mediated knock-down of NRF2 and AHR in TCDD-stimulated human foreskin keratinocytes and SPRR2 and SLPI expression in human basal cell carcinoma

(**A,B**) qRT-PCR of *NRF2* and *K14* (A) and *AHR*, *CYP1A1* and *K14* (B) relative to *GAPDH* using RNA from HFKs transfected with siRNAs targeting *NRF2* (Nrf2-2 or Nrf2-7) (A) or siRNAs targeting *AHR* (AHR1 or AHR2) (B) or scrambled (scr) siRNA (N=3/4). Cells were treated with 10^{-8}M TCDD or vehicle. Note down-regulation of *NRF2* in cells transfected with siRNAs targeting *NRF2* and of *AHR* and *CYP1A1* in cells transfected with siRNAs targeting *AHR*. No change in expression of *K14* was observed after TCDD treatment or siRNA transfection. Values are shown as the mean with SD. (**C**) Immunohistochemistry staining of SPRR2 and SLPI on sections of normal human skin

(left panel) and basal cell carcinoma (BCC) (right panel). Note strong SPRR2 and SLPI staining in differentiated keratinocytes of normal skin, but weaker staining in BCCs. Scale bar: 100μm.

		Female		Male	
		**tg/wt**	**tg/tg**	**tg/wt**	**tg/tg**
**6 month**	**Incidence**	**0%** (0/5)	**25%** (2/8)	**0%** (0/4)	**33%** (1/3)
	**Multiplicity**	**0**	**0.25**	**0**	**0.33**
**1 year**	**Incidence**	**0%** (0/12)	**27%** (3/11)	**0%** (0/10)	**71%** (5/7)
	**Multiplicity**	**0**	**0.6**	**0**	**1**
**2 years**	**Incidence**	**0%** (0/12)	**58%** (10/17)	**25%** (1/4)	**84%** (5/6)
	**Multiplicity**	**0**	**1.2**	**0.25**	**1.5**

Supporting Information Table S1: Incidence and multiplicity of macroscopically visible cysts in tail skin of 1 year- and 2 year-old female and male control and K5cre-CMVcaNrf2 mice. Cyst incidence is shown in percentage and in absolute numbers (in brackets).

**Name**	**Forward**	**Reverse**
qRT-PCR primers for mouse genes		
Ahr	ATCGCCACTCAGAGACCACT	AGGGCTGGAGATCTCGTACA
Adph	CCTCAGCTCTCCTGTTAGGC	AGGTTGGCCACTCTCATCAC
Areg	AGGCTCAGGCCATTATGCAGC	TCCCCTGTGGAGAGTTCACTGCC
