# Supplementary material for: Activation of Nrf2 in keratinocytes causes chloracne (MADISH)-like skin disease in mice
Source: EMBO Mol Med. 2014 Feb 6;6(4):442–57. doi: 10.1002/emmm.201303281 (PMC3992072; doi:10.1002/emmm.201303281)
Supplement: Supplementary file 9 [file emmm0006-0442-sd9.pdf]

(left panel) and basal cell carcinoma (BCC) (right panel). Note strong SPRR2 and SLPI staining in differentiated keratinocytes of normal skin, but weaker staining in BCCs. Scale bar: 100μm.

		Female		Male	
		tg/wt	tg/tg	tg/wt	tg/tg
**6 month**	**Incidence**	**0%** (0/5)	**25%** (2/8)	**0%** (0/4)	**33%** (1/3)
	**Multiplicity**	**0**	**0.25**	**0**	**0.33**
**1 year**	**Incidence**	**0%** (0/12)	**27%** (3/11)	**0%** (0/10)	**71%** (5/7)
	**Multiplicity**	**0**	**0.6**	**0**	**1**
**2 years**	**Incidence**	**0%** (0/12)	**58%** (10/17)	**25%** (1/4)	**84%** (5/6)
	**Multiplicity**	**0**	**1.2**	**0.25**	**1.5**

Supporting Information Table S1: Incidence and multiplicity of macroscopically visible cysts in tail skin of 1 year- and 2 year-old female and male control and K5cre-CMVcaNrf2 mice. Cyst incidence is shown in percentage and in absolute numbers (in brackets).

Name	Forward	Reverse
qRT-PCR primers for mouse genes		
Ahr	ATCGCCACTCAGAGACCACT	AGGGCTGGAGATCTCGTACA
Adph	CCTCAGCTCTCCTGTTAGGC	AGGTTGGCCACTCTCATCAC
Areg	AGGCTCAGGCCATTATGCAGC	TCCCCTGTGGAGAGTTCACTGCC
