# Supplementary material for: Activation of Nrf2 in keratinocytes causes chloracne (MADISH)-like skin disease in mice
Source: EMBO Mol Med. 2014 Feb 6;6(4):442–57. doi: 10.1002/emmm.201303281 (PMC3992072; doi:10.1002/emmm.201303281)
Supplement: Supplementary file 10 [file emmm0006-0442-sd10.pdf]

(left panel) and basal cell carcinoma (BCC) (right panel). Note strong SPRR2 and SLPI staining in differentiated keratinocytes of normal skin, but weaker staining in BCCs. Scale bar: 100μm.

| | | Female | | Male | |
|---|---|---|---|---|---|
| | | **tg/wt** | **tg/tg** | **tg/wt** | **tg/tg** |
| **6 month** | **Incidence** | **0%** (0/5) | **25%** (2/8) | **0%** (0/4) | **33%** (1/3) |
| | **Multiplicity** | **0** | **0.25** | **0** | **0.33** |
| **1 year** | **Incidence** | **0%** (0/12) | **27%** (3/11) | **0%** (0/10) | **71%** (5/7) |
| | **Multiplicity** | **0** | **0.6** | **0** | **1** |
| **2 years** | **Incidence** | **0%** (0/12) | **58%** (10/17) | **25%** (1/4) | **84%** (5/6) |
| | **Multiplicity** | **0** | **1.2** | **0.25** | **1.5** |

**Supporting Information Table S1:** Incidence and multiplicity of macroscopically visible cysts in tail skin of 1 year- and 2 year-old female and male control and K5cre-CMVcaNrf2 mice. Cyst incidence is shown in percentage and in absolute numbers (in brackets).

| Name | Forward | Reverse |
|---|---|---|
| ***qRT-PCR primers for mouse genes*** | | |
| *Ahr* | ATCGCCACTCAGAGACCACT | AGGGCTGGAGATCTCGTACA |
| *Adph* | CCTCAGCTCTCCTGTTAGGC | AGGTTGGCCACTCTCATCAC |
| *Areg* | AGGCTCAGGCCATTATGCAGC | TCCCCTGTGGAGAGTTCACTGCC |

| | | |
|---|---|---|
| *Btc* | CCAATGGCTCTCTTTGTGGA | CCGAGAGAAGTGGGTTTTCA |
| *DNA_ns* | GCAGCACAAGGAACATTCAG | CGCCTAACTGCAGGTGATGT |
| *Egf* | TCGAGAGAAGCGAGAGAAGC | CACCAATTGCTGGTGATTTG |
| *Epgn* | ATGAGCTGAAGCAAGCCATT | CTCCCTCCAGAGCAGATGAT |
| *Epgn_ARE* | TGAAATCAATGGATGAGGATG | TGCCCTGATGCTAAGGTTTC |
| *Epgn_prom* | CAGCAGGGAATTTTCCACAT | GGCACTCGCACACTTGTTTA |
| *Ereg* | TATCAGCACAACCGTGATCC | GGGATCGTCTTCCATCTGAAC |
| *Gapdh* | TCGTGGATCTGACGTGCCGCCTG | CACCACCCTGTTGCTGTAGCCGTAT |
| *Gclc* | AACAAGAAACATCCGGCATC | CGTAGCCTCGGTAAAATGGA |
| *Gclm* | TCCCATGCAGTGGAGAAGAT | AGCTGTGCAACTCCAAGGAC |
| *Gsta3* | TACTTTGATGGCAGGGGAAG | GCACTTGCTGGAACATCAGA |
| *Hbegf* | TAGCTCCCACTGAGGAGGAC | TCATGGCTGCTGGTGAAATA |
| *Lce3a* | CCTATCACCTTCCCATCTGGT | GCTGGCACTGCTTCTGACTC |
| *Mc5r* | CAGAGCCCGGTAAACAGAAG | CACTTCTTGGCATCGGATTT |
| *Nqo1* | CTGGCCCATTCAGAGAAGAC | GTCTGCAGCTTCCAGCTTCT |
| *Nqo1_ARE* | AGCAGAACGCAGCACGAAT | CACTCAGCCGTGGGAAGT |
| *Nqo1_ns* | TACGCTGTAGTGGTGGTGGA | TCTGGGGACTTGGGTATCTG |
| *Nrf2* | CCAGCTACTCCCAGGTTGC | CCAAACTTGCTCCATGTCCT |
| *Plin2 (Adph)* | CCTCAGCTCTCCTGTTAGGC | AGGTTGGCCACTCTCATCAC |
| *Pparg* | TGATTACAAATATGACCTGAAGCTCC | TTGTAGAGCTGGGTCTTTTCAGAA |
| *S100A7* | CTTGTCCCTGGAGGAGTTGA | GCAGATCTGGTTGTCCTTGTT |
| *Scd1* | CGGTGCACAACGTACCAAT | TTGATCCAGAGGACGTCGAT |
| *Scd2* | GTCGCTGAGGTCTGAAGCTC | TTGTGGTGGTGGCTGAGTAA |
| *Scd3* | CTGCAAGAAGAGATGACGCC | GTAGACAAACGCGAAGAGGC |
| *Scd4* | TCTAGGCTTCCAAGGAGCAA | TTCAGGCCGGATATCTTCTG |
| *Slpi* | GGGCAAATACAAGTGCTGTG | CCTGGGAGCAGGGAAGTAGT |

| | | |
|---|---|---|
| *Sprr2a* | TGAGGCAGGCAATCCTATAAA | GATTGCACTGCTGCTGGTAG |
| *Sprr2d* | CTGGTACTCAAGGCCGAGAC | CAGGGCACTTTGGTGGAG |
| *Srxn1* | CGGTGCACAACGTACCAAT | TTGATCCAGAGGACGTCGAT |
| *Tgfa* | CTGAGTGACTCACCCGTGGC | GCGGAGCTGACAGCAGTGGAT |
| ***qRT-PCR primers for human genes*** | | |
| *AHR* | ATCCAGTACTGCCAGGCCAA | AAGGTCTGGCTTCTGACGGA |
| *CYP1A1* | CCAGCTCAGCTCAGTACCTCA | AAGATGACAGAGGCCAGAAGA |
| *CYP1B1* | AACGTACCGGCCACTATCAC | GCACTCGAGTCTGCACATCA |
| *GAPDH* | AAGGTCGGAGTCAACGGATT | CTCCTGGAAGATGGTGATGG |
| *GCLC* | ACTCCCTCATCCATCTGGCAA | ACTCCCTCATCCATCTGGCAA |
| *EPGN* | TGACAGCACTGACCGAAGAG | CCAGGCAAAGGTGTGAGAAC |
| *K14* | AGGAGGAGATGAATGCC | AGCTCCTCTGTCTTGGTGAAG |
| *NQO1* | GTGATATTCCAGTTCCCCCTGC | AAGCACTGCCTTCTTACTCCGG |
| *NRF2* | AGGTTGCCCACATTCCCAAA | AATGTCTGCGCCAAAAGCTG |
| *SLPI* | CTGTGGAAGGCTCTGGAAAG | CAGTCACTCTGGCACTCAGG |
| *SRXN1* | CAAGGTGCAGAGCCTCGT | GATGGTCTCTCGCTGCAGTT |
| *SPRR2D* | CTTTCTCCTTAACCTGTGGCCT | GCAGTATGGCAGCCTCAGA |
| ***siRNAs*** | | |
| *AHR1* | GGCUCUUUCAAGAUAGUAA [dTdT] | |
| *AHR2* | CGGAUGAAAUCCUGACGUA [dTdT] | |
| *Nrf2-2* | GAGAAAGAAUUGCCUGUAA [dTdT] | |
| *Nrf2-7* | GCUCAUACUUUAUAAGUAA [dTdT] | |
| ***Primers for cloning of in situ hybridization probes*** | | |
| *Epgn-T7* | taatacgactcactatagggATGTTGAGTGCCCGAAGAAC | |
| *Epgn-SP6* | gatttaggtgacactatagCCTAGCACAGCACAGCAGAG | |

**Supporting Information Table S2: List of oligonucleotides and their sequences**
