# Supplementary material for: Activation of Nrf2 in keratinocytes causes chloracne (MADISH)-like skin disease in mice
Source: EMBO Mol Med. 2014 Feb 6;6(4):442–57. doi: 10.1002/emmm.201303281 (PMC3992072; doi:10.1002/emmm.201303281)
Supplement: Supplementary file 11 [file emmm0006-0442-sd11.pdf]

SUPPORTING INFORMATION related to the article

Activation of Nrf2 in keratinocytes causes chloracne (MADISH)-like skin disease in mice

Matthias Schäfer, Ann-Helen Willrodt, Svitlana Kurinna, Andrea S. Link, Hany Farwanah, Alexandra Geusau, Florian Gruber, Olivier Sorg, Aaron J. Huebner, Dennis R. Roop, Konrad Sandhoff, Jean-Hilaire Saurat, Erwin Tschachler, Marlon R. Schneider, Lutz Langbein, Wilhelm Bloch, Hans-Dietmar Beer, and Sabine Werner

TABLE OF CONTENTS

Supporting Information Figures and Tables …….. 2
Supporting Information Fig S1 ..…………………………………………………....... 2
Supporting Information Fig S2 ………………...………..…………………………....... 4
Supporting Information Fig S3 ……………………...………................................. 5
Supporting Information Fig S4 …………………………….....……………………....... 6
Supporting Information Fig S5 ……………………………………………………… 7
Supporting Information Fig S6 ………………………………………........................... 8
Supporting Information Fig S7 ………………………………........………………… 9
Supporting Information Table S1 ……………..……………........………………........ 10
Supporting Information Table S2 …………………………...…..………………........ 11
